# The Role of Glypicans in Wnt Inhibitory Factor-1 Activity and the Structural Basis of Wif1's Effects on Wnt and Hedgehog Signaling

**DOI:** 10.1371/journal.pgen.1002503

**Published:** 2012-02-23

**Authors:** Andrei Avanesov, Shawn M. Honeyager, Jarema Malicki, Seth S. Blair

**Affiliations:** 1 Department of Zoology, University of Wisconsin, Madison, Wisconsin, United States of America; 2 Genetics Training Program, University of Wisconsin, Madison, Wisconsin, United States of America; 3 Division of Craniofacial and Molecular Genetics, Tufts University, Boston, Massachusetts, United States of America; Harvard Medical School, Howard Hughes Medical Institute, United States of America

## Abstract

Proper assignment of cellular fates relies on correct interpretation of Wnt and Hedgehog (Hh) signals. Members of the Wnt Inhibitory Factor-1 (WIF1) family are secreted modulators of these extracellular signaling pathways. Vertebrate WIF1 binds Wnts and inhibits their signaling, but its *Drosophila melanogaster* ortholog Shifted (Shf) binds Hh and extends the range of Hh activity in the developing *D. melanogaster* wing. Shf activity is thought to depend on reinforcing interactions between Hh and glypican HSPGs. Using zebrafish embryos and the heterologous system provided by *D. melanogaster* wing, we report on the contribution of glypican HSPGs to the Wnt-inhibiting activity of zebrafish Wif1 and on the protein domains responsible for the differences in Wif1 and Shf specificity. We show that Wif1 strengthens interactions between Wnt and glypicans, modulating the biphasic action of glypicans towards Wnt inhibition; conversely, glypicans and the glypican-binding “EGF-like” domains of Wif1 are required for Wif1's full Wnt-inhibiting activity. Chimeric constructs between Wif1 and Shf were used to investigate their specificities for Wnt and Hh signaling. Full Wnt inhibition required the “WIF” domain of Wif1, and the HSPG-binding EGF-like domains of either Wif1 or Shf. Full promotion of Hh signaling requires both the EGF-like domains of Shf and the WIF domains of either Wif1 or Shf. That the Wif1 WIF domain can increase the Hh promoting activity of Shf's EGF domains suggests it is capable of interacting with Hh. In fact, full-length Wif1 affected distribution and signaling of Hh in *D. melanogaster*, albeit weakly, suggesting a possible role for Wif1 as a modulator of vertebrate Hh signaling.

## Author Summary

In developing organisms, cells choose between alternative fates in order to make appropriately patterned tissues, and misregulation of those choices can underlie both developmental defects and cancers. Cells often make these decisions because of signals received from neighboring cells, such as those mediated by the secreted signaling proteins of the Wnt and Hedgehog (Hh) families. While signaling can be regulated by the levels of signaling or receptor proteins expressed by cells, another level of control is exerted by proteins that bind signaling proteins outside of cells and either inhibit or promote the signaling process. In the fruitfly *Drosophila* *melanogaster*, the secreted Shifted protein has been shown to bind Hh and to increase Hh signaling, likely by reinforcing interactions between Hh and cell surface proteins of the glypican family. We provide evidence that the vertebrate homolog of Shifted, Wnt Inhibitory Factor-1 (Wif1), inhibits Wnt activity by a similar mechanism, reinforcing interactions between Wnts and glypicans in a manner that sequesters Wnts from their receptors. We also examine the structural basis for the specificities of Wif1 and Shifted for Wnt and Hh signaling, respectively, and provide evidence that Wif1, although a potent inhibitor of Wnt activity, influences *D. melanogaster* Hh signaling.

## Introduction

The extracellular space provides an important milieu for the regulation of signaling by Wnt and Hedgehog (Hh) morphogens. Several factors are known that bind secreted Wnts or Hhs and regulate either their extracellular levels, their movement through tissues, or their access to receptors. Members of the Wnt Inhibitory Factor-1 (WIF1) family of secreted proteins are unusual, however, because they can impact either the Wnt or Hh pathways. Vertebrate WIF1 binds Wnts and inhibits Wnt signaling [1], while the *Drosophila melanogaster* WIF1 homolog Shifted (Shf, NCBI Gene ID: 31617) binds Hh and promotes Hh signaling [2], [3]. This study investigates the mechanism of vertebrate WIF1 action, and the basis of the different activities of the vertebrate and *D. melanogaster* WIF1 family proteins.

Human WIF1 (NCBI Gene ID: 11197) binds vertebrate Wnts and the *D. melanogaster* Wnt Wingless (Wg, NCBI Gene ID: 34009) and, in gain-of-function assays, WIF1 inhibits vertebrate Wnt and *D. melanogaster* Wg signaling [1], [3]–[7]. Morpholino-induced knockdown of *wif1* in zebrafish (*Danio rerio*) embryos results in shortening along the anterior-posterior axis, defective somites and increased canonical Wnt signaling in the developing swimbladder [8]. Blocking WIF1 function also increases rod production in cultures of dissociated rat retinas, similar to the effects of increasing Wnt4 signaling [6]. And while knocking out *Wif1* in mice does not lead to obvious developmental defects, it does increase the growth of radiation-induced osteosarcomas [9]. Human *WIF1* is also epigenetically silenced in many tumors that have heightened Wnt signaling, and addition of exogenous WIF1 to such tumors reduces Wnt signaling, slows tumor growth and increases apoptosis [9]–[20].

Shf is the only *D. melanogaster* member of the WIF1 family but, unlike vertebrate WIF1, Shf cannot inhibit Wg signaling. Instead, Shf binds Hh (NCBI Gene ID: 42737), and loss of Shf reduces both the accumulation of extracellular Hh and the range of Hh signaling in the *D. melanogaster* wing disc [2], [3]. Shf appears to mediate these effects by stabilizing interactions between Hh and the glypican family of membrane-bound Heparan Sulfate Proteoglycans (HSPGs). Glypicans are anchored to the cell surface by glycosylphosphatidylinositol (GPI) linkages, and regulate signaling by binding a variety of signaling and signal-binding molecules, including Hh and Shf [2], [21], [22]. Removing the two *D. melanogaster* glypicans, Dally (NCBI Gene ID: 39013) and Dally-like protein (Dlp, NCBI Gene ID: 39596), or blocking synthesis of their HS glycosaminoglycan sidechains, reduces the extracellular accumulation of extracellular Shf [2]; binding between HS and WIF1 family members is direct [7]. Loss of *dally* and *dlp* or HS synthesis in *D. melanogaster* wing discs mimics the loss of Shf, similarly reducing the accumulation of extracellular Hh and the range of Hh signaling in wing discs [23]–[28]. Thus, the interaction between Hh and the glypicans appears to be weakened or eliminated by the loss of Shf; conversely, Shf function depends in large part on the presence of the glypicans [3].

While binding has been demonstrated between HS and vertebrate WIF1 [7], the function of this binding is unknown. An important question is therefore whether (and how) HSPGs contribute to the Wnt-inhibiting functions of vertebrate WIF1. Glypicans have complex effects on Wnt signaling [21], [22], [29]. In some contexts, the loss of glypicans reduces Wnt signaling, consistent with a co-receptor-like role, or a less direct effect on Wnt accumulation or movement; however, in other contexts the loss of glypicans increases Wnt signaling, suggesting that glypicans can sequester Wnts away from their receptors [29]–[37]. Indeed, the ability of both *D. melanogaster* and vertebrate glypicans to promote or inhibit Wnt signaling is “biphasic”, depending in part on their concentration; low levels promote and high levels inhibit [30], [35], [38], [39] (see Discussion).

We will provide evidence that in at least two contexts, the exogenous assay provided by the wing disc of *D. melanogaster*, and the early embryo of zebrafish, the inhibitory activity of the zebrafish WIF1 homolog (Wif1, Entrez Gene ID: 30476) is greatly facilitated by its ability to act as a bridge between Wnts and glypicans. In this sense, Wif1 can bias the biphasic activity of glypicans, increasing their ability to inhibit Wnt signaling.

We have also examined the structural basis for this interaction. All WIF1 family members (including Shf) are composed of two distinct regions. At the N-terminal end is the Wnt-binding ‘WIF’ domain [1]. This is followed by five ‘EGF-like’ domains; we will provide evidence that these are required for interactions between Wif1 and glypicans, consistent with recent biochemical data [7].

Finally, given the similarities between Wif1 and Shf, what controls their pathway specificity, and does vertebrate Wif1 have any overlapping activity in the promotion of Hh activity? To answer these questions we swapped domains between Wif1 and Shf. Our results show that the ‘EGF-like’ domains, but not the ‘WIF’ domains, of Wif1 and Shf are largely interchangeable for the inhibition of Wnt signaling. The ‘EGF-like’ domains are not, however, interchangeable for the promotion of Hh signaling, while the ‘WIF’ domains are. We will also show that Wif1 can affect the accumulation and, weakly, the activity of *D. melanogaster* Hh, suggesting that vertebrate WIF1 proteins have the potential to regulate vertebrate Hh signaling.

## Results

### Zebrafish Wif1 inhibits Wg signaling in *D. melanogaster*

**Figure 1:**
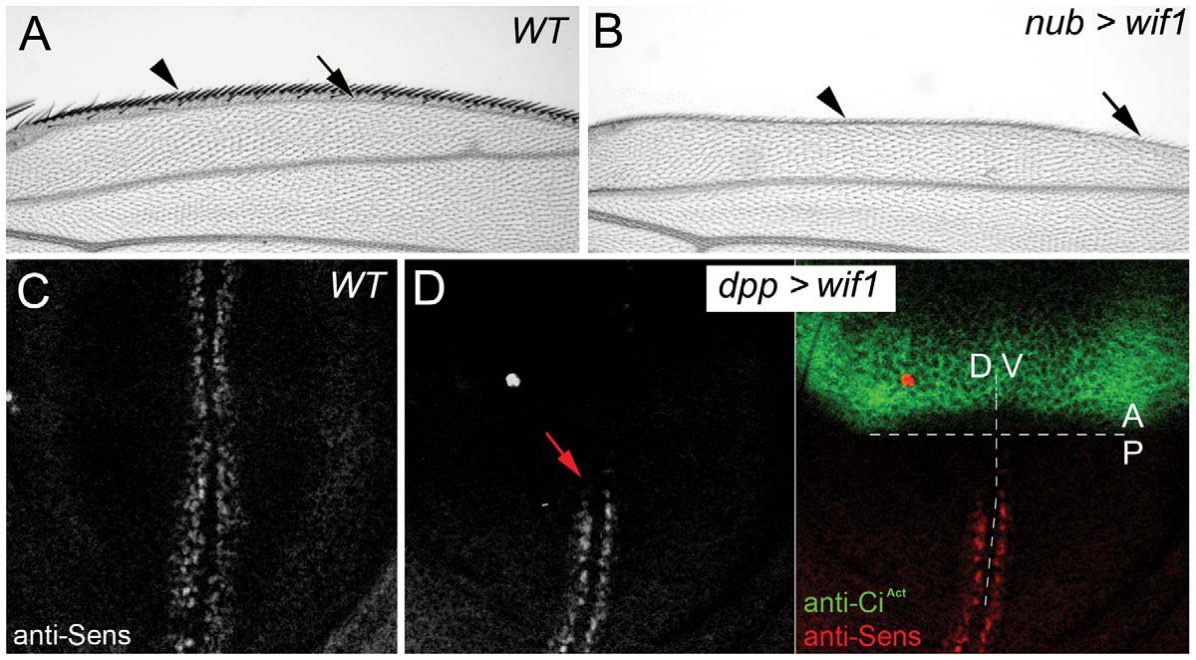
Zebrafish Wif1 inhibits *D. melanogaster* Wg. (A) Anterior margin of wild-type (WT) wing shows a dense array of sensory bristles (arrowhead). First longitudinal vein (L1, arrow) marks the anterior edge of the wing blade. (B) *nub-Gal4*-driven expression of *UAS-wif1* eliminates anterior bristles (arrowhead) and disrupts L1 (arrow). (C) Anti-Sens staining along the presumptive wing margin in wild type late third instar wing disc. (D) *dpp-Gal4*-driven expression of *UAS-wif1* in anterior cells of late third instar wing disc (marked green by anti-Ci^Act^) eliminates anti-Sens staining locally and in adjacent posterior cells (arrow). In these and the remaining figures anterior is up. In adult wings distal is to the right, in wing discs ventral is to the right.

In order to analyze the function of Wif1, we first made use of the in vivo assays and genetic manipulations provided by the developing wing of *D. melanogaster*. Three *D. melanogaster* Wnt family members, Wg, Wnt4 (NCBI Gene ID: 34007) and Wnt6 (NCBI Gene ID: 34010), are co-expressed in a narrow stripe of cells along the prospective wing margin in mid-to-late third instar wing discs, but Wnt4 and Wnt6 are not known to affect wing margin development [40]–[43]. Wg is, however, necessary and sufficient for the development of dorsal and ventral rows of sensory and non-sensory bristles that arise adjacent to the Wnt-expressing cells; strong loss of Wg signaling also eliminates more proximal tissues, leading to reduced wings with a scalloped margin [44]–[46] (Figure S1). Driving expression of *UAS-wif1* with the wing blade driver *nubbin-Gal4* (*nub-Gal4*) produced adult wing phenotypes indicative of reduced Wg activity (compare Figure 1A, 1B to Figure S1). In wing discs, distal Wg induces adjacent dorsal and ventral rows of anti-Senseless (Sens) staining (Figure 1C) [47], [48], and we found that driving expression of *UAS-wif1* with *dpp-Gal4*, whose expression is limited to anterior cells near the compartment boundary [49], eliminated anti-Sens staining not only in the anterior, and also non-autonomously in nearby posterior cells, indicating that the secreted Wif1 can act over several cell diameters (Figure 1D). Thus, zebrafish Wif1 can inhibit signaling by the *D. melanogaster* Wnt Wg, much like human WIF1 [1], [3].

### Wif1 increases the accumulation of extracellular Wg on Dlp-expressing cells

**Figure 2:**
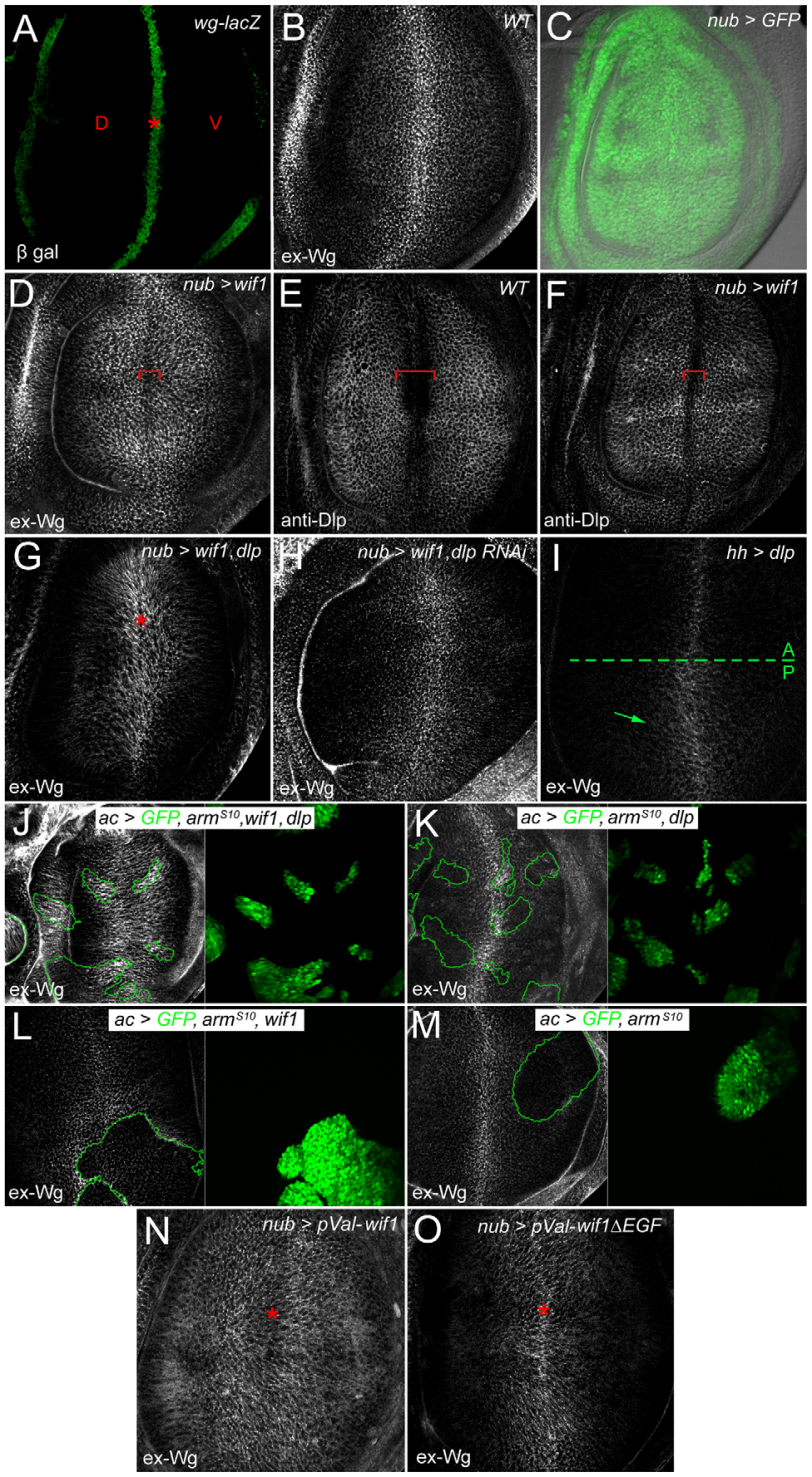
Wif1 stabilizes Wg on Dlp-expressing cells in late third instar wing discs. Wing pouch regions of wing imaginal discs. (A) *wg-lacZ* expression along the prospective wing margin (asterisk) where prospective dorsal (D) and ventral (V) wing blade surfaces abut. (B) Extracellular Wg (ex-Wg) from the *wg*-expressing cells, which is high distally and lower proximally. (C) Pattern of *nub-Gal4* expression, marked by *UAS-GFP*. In all subsequent panels, except for I-M, *nub-Gal4* is used to drive UAS-transgene expression. (D) ex-Wg after expression of *UAS-wif1*. ex-Wg is higher on proximal cells than on distal ones (red bracket). (E) Anti-Dlp staining in wild-type wing disc. Dlp expression is downregulated in distal cells of the prospective wing margin (red bracket). (F) Anti-Dlp staining after *UAS-wif1* expression. The width of the prospective wing margin region with reduced staining (red bracket) is narrowed compared to anti-Dlp staining in the wild-type disc in E. (G) ex-Wg staining after co-expression of *UAS-wif1* and *UAS-dlp*. ex-Wg is increased at the wing margin (asterisk). (H) ex-Wg staining after co-expression of *UAS-wif1* and *UAS-dlp RNAi* is similar to that in the wild-type disc in E. (I) Posterior expression of *UAS-dlp* (using *hh-Gal4*). ex-Wg accumulates in the posterior compartment. (J-M) Flpout *actin-Gal4* (*ac*) clones marked with *UAS-GFP* (green). (J) High ex-Wg levels inside and, to a lesser extent, outside clones expressing: *UAS-arm^S10^*, *UAS-wif1*, and *UAS-dlp*. (K) Low, largely unchanged ex-Wg levels inside clones expressing *UAS-arm^S10^* and *UAS-dlp*. (L) Reduced ex-Wg levels in clones expressing *UAS-wif1* and *UAS-arm^S10^*. zWIF1 increases ex-Wg outside the clone. (M) Reduced ex-Wg levels in clones expressing Arm^S10^. (N) After expression of *pVal-UAS-wif1*, ex-Wg staining is high proximally and low along the wing margin (asterisk). (O) Expression of *pVal-UAS-wif1*Δ*EGF* does not lead to a strong increase in proximal ex-Wg, and does not decrease ex-Wg along the wing margin (asterisk).

Conventional anti-Wg staining suggests that expressing human WIF1 in wing discs reduces Wg internalization, perhaps by reducing receptor-mediated endocytosis [3]. Little is known, however, about how WIF1 affects extracellular Wg, which is poorly visualized by conventional staining. We therefore used an alternate method that stains extracellular Wg (ex-Wg), and that reveals a gradual gradient of ex-Wg in wild type discs from the distal, *wg*-expressing cells to proximal cells that lack *wg* expression (Figure 2A, 2B) [50].

When we used *nub-Gal4* to express zebrafish *wif1* throughout the presumptive wing blade (Figure 2C), the pattern of wild-type ex-Wg distribution was inverted: ex-Wg levels were greatly elevated proximally, and distal levels near the prospective wing margin were reduced (compare Figure 2D, 2B). The Wif1-induced ex-Wg pattern was strikingly similar to the distribution of the glypican Dlp, which is high within the wing pouch but reduced along the prospective wing margin (Figure 2E) [33], [35]. Although the region of reduced ex-Wg at the margin of *wif1*-expressing discs was somewhat narrower than the normal Dlp-deficient zone of wild-type discs, anti-Dlp staining also revealed that the zone with diminished Dlp was narrower in *nub-Gal4*, *UAS-wif1* discs (Figure 2F). This change in Dlp expression is most likely a consequence of the reduced Wg activity caused by Wif1; a previous study showed that Wg signaling downregulates Dlp levels [33]. Thus, the distribution of ex-Wg after *wif1* expression strongly resembles that of Dlp.

To test the role of Dlp in the distribution of ex-Wg, we first co-expressed *dlp* and *wif1* using *nub-Gal4*, and found that ex-Wg now accumulated on the cells of the presumptive wing margin, likely because of the ex-Wg is bound by high levels of distal Dlp (Figure 2G). Next, we simultaneously expressed *wif1* and knocked-down endogenous Dlp levels. In *nub-Gal4*, *UAS-wif1*, *UAS-dlp RNAi* discs, the ex-Wg gradient reverted (Figure 2H) to resemble the wild-type ex-Wg gradient (e.g. Figure 2B). Thus, Dlp is both sufficient and necessary for much of the Wif1-induced redistribution of ex-Wg.

The above data indicates that Wif1 increases the levels of Dlp-bound Wg, thereby increasing the accumulation of ex-Wg on cell surfaces that have high levels of Dlp. We hypothesize that this also reduces the levels of free, diffusible Wg around proximal cells, creating a diffusion “sink” that in turn reduces the levels of Wg around the distal, Wg-secreting cells where Dlp levels are low.

However, since Wg signaling can reduce Dlp levels [33], and Dlp stabilizes Wg [33], [51], [52], it is possible that *wif1* expression increases Dlp levels (and thus Dlp-bound Wg) by inhibiting Wg signaling. Our initial results argue against this, since *wif1* expression did not obviously affect anti-Dlp staining, except by narrowing the zone with low Dlp levels near the wing margin (Figure 2F). As a more rigorous test we examined the effects of *wif1* expression on ex-Wg in cells with fixed levels of Wg signaling and *dlp* transcription. We drove Wg signaling at high levels by expressing Armadillo (Arm)^S10^, a constitutively active, Wnt-independent form of the *D. melanogaster* β-Catenin Arm (NCBI Gene ID: 31151) [53], and drove *dlp* transcription at high levels using *UAS-dlp*. To bypass the deformation of wing tissues expected from widespread Arm^S10^ expression, we used the Gal4 Flpout technique to generate clonal clusters of cells misexpressing *UAS-arm^S10^*, either alone or in combination with *UAS-dlp* and/or *UAS-wif1*. First, we found that ex-Wg levels were much higher within clones expressing *UAS-arm^S10^*, *UAS-wif1* and *UAS-dlp* than in clones expressing only *UAS-arm^S10^* and *UAS-dlp* (compare clones in Figure 2J, 2K). As expected from the diffusion of Wif1, ex-Wg also accumulated outside the *UAS-wif1*, *UAS-arm^S10^ UAS-dlp* clones, albeit not at levels quite as high as inside the clone. These results indicate that Wif1 increased the accumulation of ex-Wg independently of any effects that may have been caused by the repression of Wg signaling. Ex-Wg levels were reduced in clones co-misexpressing *UAS-arm^S10^* and *UAS-wif1* (Figure 2L) or *UAS-arm^S10^* alone (Figure 2M). This is most likely due to the reduction of endogenous Dlp levels by Arm^S10^ [33], and is consistent with the requirement for Dlp in Wif1-dependent stabilization of ex-Wg (see Figure 2J).

Since WIF1 binds directly to HS and Wnts [7], we propose that Wif1 stabilizes or reinforces the binding between glypican HSPGs and Wnt on the cell surface. This parallels the role proposed for the *D. melanogaster* Wif1 homolog Shf, which binds Hh and glypicans and is thought to thereby stabilize Hh on cell surfaces [2], [3]. However, in the case of Shf the increased Hh accumulation is accompanied by increased Hh signaling. It was striking that the accumulation of ex-Wg around proximal cells caused by *wif1* expression was not accompanied by obvious gains in proximal Wg signaling, since ectopic anti-Sens staining or bristle development in proximal cells was never observed (Figure 1), nor could we detect obvious effects on the low-level Wg target Distal-less (data not shown). The reduction of distal ex-Wg levels caused by *wif1* expression might explain the reduced Wg signaling at the presumptive margin. However, distal co-expression of *dlp* and *wif1* increases ex-Wg accumulation at the presumptive wing margin (Figure 2G), yet we will show in the following section that this leads to an even stronger reduction of distal Wg signaling. Thus, ex-Wg depletion from the presumptive wing margin is unlikely to be responsible for the defects in wing margin signaling. Rather, the ex-Wg that accumulates at high levels on the surfaces of Dlp-expressing cells is incapable of activating Wg receptors.

This data shows a strong role for Wif1 and Dlp in Wg accumulation, but does not rule out a partially redundant role for the other *D. melanogaster* glypican, Dally. *dally* is transcribed at slightly higher levels along the distal margin [33], [54], which would not be consistent with the pattern of extracellular Wg accumulation induced by *wif1* expression. However, since there are no antisera to Dally its pattern of extracellular accumulation is unknown. Evidence suggests that Dally's levels may actually be reduced at the margin: the GPI-linkage between Dally and cell membranes might be cleaved along the distal wing margin by distally-expressed Notum, as suggested both by genetic interactions [33] and the modification of Dally by Notum in vitro [51], although overexpressed Dally-HA accumulates uniformly in wing discs [35]. Below we will show genetic interactions consistent with redundant activities of both glypicans.

### Glypicans promote Wif1-dependent inhibition of Wnt signaling

**Figure 3:**
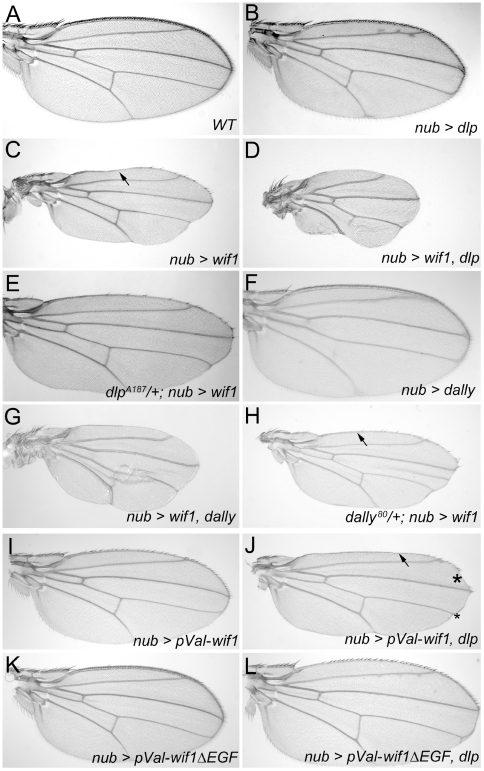
Dlp and Dally enhance the effects of Wif1 expression in *Drosophila* wings. (A–L) *nub-Gal4* is used to drive transgene expression. (A) Wild-type wing. (B) Overexpression of *UAS-dlp* results in only slightly fewer bristles along the wing margin, no loss of L1 and no reduction in wing size. For a detailed comparison of bristle numbers, see Figure S3. (C) Expression of *UAS-wif1* eliminates many bristles, interrupts L1 (arrow) and somewhat reduces wing size. (D) Co-expression of *UAS-dlp* and *UAS-wif1* almost completely eliminates wing margin bristles and L1, and strongly reduces wing size. (E) Expression of *UAS-wif1* causes much weaker wing margin defects in *dlp^A187^/+* heterozygotes. (F) Overexpression of *UAS-dally* results in only very slightly fewer wing margin bristles and no obvious reductions in wing size. For detailed comparison of bristle numbers see Figure S3. (G) Combined expression of *UAS-dally* and *UAS-wif1* almost completely eliminates wing margin bristles and L1, and further reduces wing size. (H) Expression of *UAS-wif1* causes weaker wing margin defects in *dally^80^*/+ heterozygotes (e.g. more complete anterior L1; compare arrows in C and H). (I–L) EGF-depleted Wif1 is less effective at inhibiting Wg signaling and does not interact with Dlp. Control wings expressing *pVal-UAS-wif1* show modest defects in margin development (I), which is synergistically enhanced by *UAS-dlp*; the arrow marks the interruption of L1 and the asterisks mark scalloping of the margin (J). *pVal-UAS-wif1*Δ*EGF* is less effective at reducing number of margin bristles than *pVal-UAS-wif1* and its effects on wing margin development are not enhanced by co-expression of *UAS-dlp* (J). See Figure S3 for comparison of bristle numbers.

We next asked how glypicans modulate the effects of Wif1 on Wnt signaling, testing first the effects of *dlp* overexpression. In control, *nub-Gal4*, *UAS-dlp* wings we found a very slight reduction of margin bristles compared to wild type (Figure S3), but wing blades were largely of normal size and showed no signs of scalloping at the margin (Figure 3A and 3B). Nonetheless, expression of *UAS-dlp* strongly enhanced the effects of a moderately strong *UAS-wif1* genomic insertion, increasing the extent of wing scalloping and bristle loss (Figure 3C, 3D). We next re-tested this interaction using a weaker *UAS-wif1* construct inserted into a viral integrase site in the genome (*pVal-UAS-wif1*). *nub-Gal4*-driven expression of *pVal-UAS-wif1* did not cause any margin scalloping or wing blade reduction on its own, but did so when co-misexpressed with *UAS-dlp* (Figure 3I, 3J and Figure S3). This indicates that the genetic interaction between *dlp* and *wif1* was not simply additive, but synergistic.

To test whether the genetic interaction between *dlp* and *wif1* was specific, we examined the effects of co-misexpressing *UAS-dlp* with a different extracellular Wg inhibitor, a GPI-linked extracellular fragment of the *D. melanogaster* Frizzled 2 (DFz2, NCBI Gene ID: 40090) Wnt receptor (*UAS-Dfz2-GPI*) that binds ex-Wg but cannot transduce Wg signal [55]. The effects of *nub-Gal4*-driven *UAS-Dfz2-GPI* expression were not strengthened by co-expression of *UAS-dlp* (Figure S2C, S2E). Dfz2-GPI-expressing wings were still sensitive to further reductions in Wg function, however, since co-expression with *UAS-wif1* reduced their size (Figure S2D).

We next examined whether reducing Dlp levels altered the effects of *wif1* expression. Since *dlp* null mutants are lethal and defective in several signaling pathways, we instead reduced Dlp levels using *dlp* heterozygotes or expression of *UAS-dlp RNAi*. In both cases, the effects of *nub-Gal4*-driven expression of the moderately strong *UAS-wif1* insertion were greatly decreased; scalloping of the adult wing margin was almost completely eliminated and more margin bristles were retained (Figure 3E and data not shown). These results demonstrate that the glypican Dlp increases the effectiveness of Wif1.

We also found similar genetic interactions between Wif1 and the *D. melanogaster* glypican Dally. Overexpression of Dally alone did not induce wing margin scalloping, and had weaker effects on bristle number than overexpression of Dlp (Figure 3F; quantified in Figure S3), in agreement with weaker effects of Dally overexpression on extracellular Wg [33], [50]. Nonetheless, Dally overexpression synergized the effects of Wif1 expression, causing additional scalloping and bristle loss (Figure 3G). Moreover, while removing *dally* weakens Wg signaling along the wing margin [36], the reduction of Wg signaling observed after Wif1 expression was partially reversed in a *dally* heterozygote background (Figure 3H). This suggests that Wif1 binds to more than one kind of HSPG, consistent with the observed interactions between WIF1 family members and the HS sidechains attached to all glypicans [2], [7]

To test if similar relationships exist between Wif1 and glypicans in the context of zebrafish Wnt signaling, we analyzed genetic interactions between Wif1 and zebrafish Glypican 4 (Gpc4, also known as Knypek, NCBI Gene ID: 118437), which is similar to Dally and Dlp and interacts with zebrafish Wnts and Wnt-binding proteins [38], [56]. WIF1 binds Wnts that stimulate both canonical β-catenin-mediated and non-canonical planar cell polarity (PCP) signaling [1], [7], [57], and WIF1 overexpression inhibits canonical Wnt signaling in several contexts [1], and PCP in the rat inner ear [58]. We found that injection of *wif1* mRNA intro zebrafish embryos inhibited canonical Wnt signaling, as indicated by the expression of the *Tg(TOP:dGFP)* reporter line [59] in the dorsal hindbrain (Figure S4). *wif1* injection also inhibited posterior development (Figure S4), phenocopying the posterior defects caused by reduced canonical signaling [56], [60]–[62], or by reduced PCP signaling and the resultant defects in convergent-extension movements [38], [63]–[67]. The most extreme phentoypes included slightly enlarged forebrains, indicative of decreased canonical signaling, but not the enlarged heads caused by very strong reductions in canonical signaling. While defects in posterior development and convergent extension movements can also be caused by changes in BMP signaling [68], [69], WIF1 does not interact with BMP signaling in frog embryos [1]. *wif1* expression in zebrafish did not induce the ventral fin defects typical of BMP-regulated changes in the dorsal-ventral axis [70], and in *D. melanogaster* Wif1 did not induce the changes in wing vein development typical of altered BMP signaling [71]. While we show below the Wif1 can affect *D. melanogaster* Hh signaling, our *wif1*-injected embryos also do not resemble those with altered Hh signaling [72], [73]. We therefore used shortening of the posterior as a measure of Wnt inhibition.

**Figure 4:**
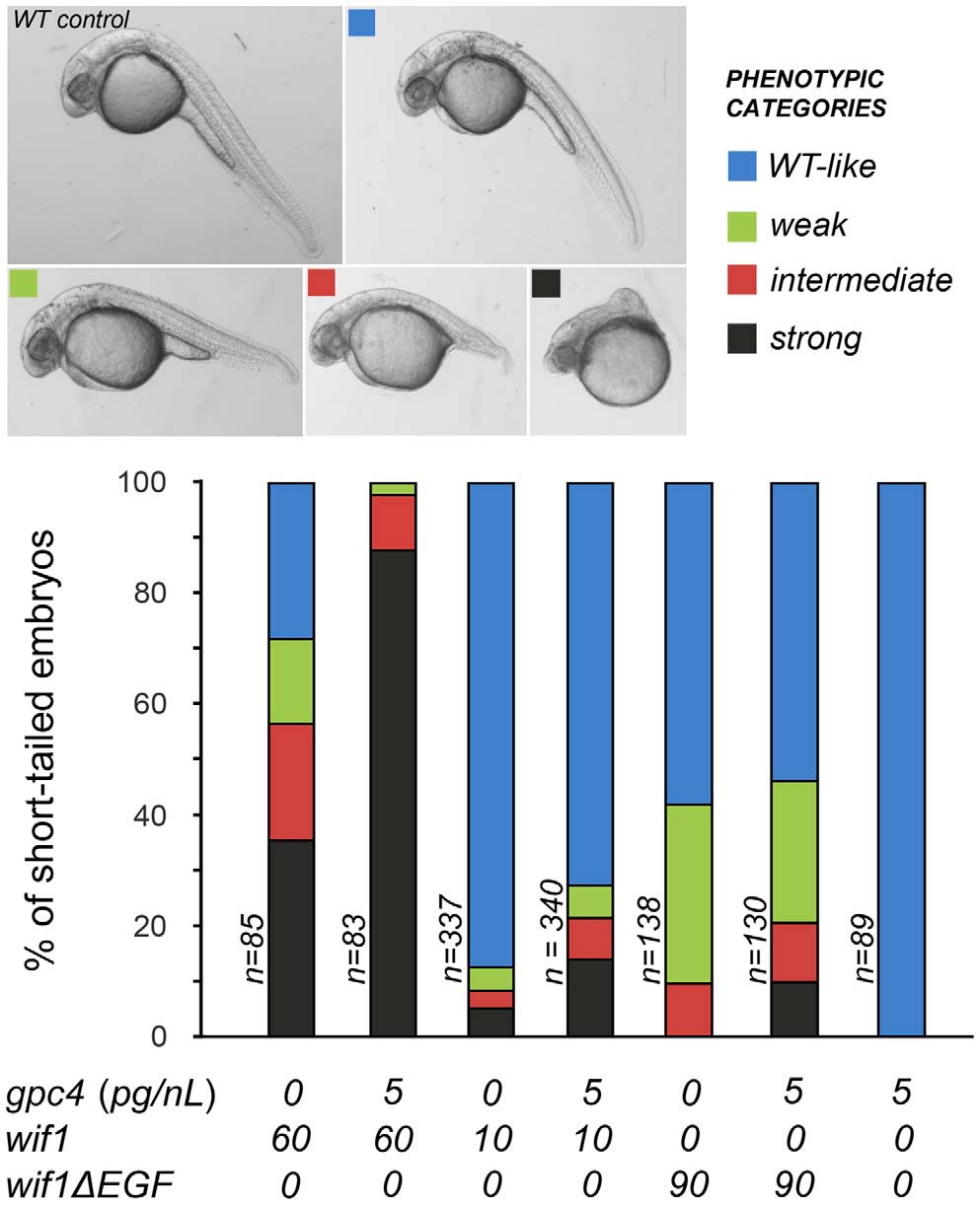
Gpc4 enhances the effects of full-length Wif1 in zebrafish embryos. Approximately 2 nL of mRNA of a given concentration was injected into one cell stage embryos, and embryos were scored at 28–30 hours post-fertilization. Images show representative examples of the penetrance of the short-tailed phenotype compared to an uninjected control. In top panels dorsal is up and anterior is to the left. Bar graphs show percentage of short-tailed embryos. The data is pooled from two independent experiments; frequencies were scored and their percentages were averaged. See Materials and Methods for information on RNA preparation.

We observed synergy between the effects of overexpressing *wif1* and *gpc4*. Control embryos injected with low (5 pg/nL) doses of *gpc4* message were morphologically indistinguishable from wild-type individuals and showed normal levels of canonical TOP:dGFP expression in the hindbrain (Figure 4 and Figure S4). As shown by others, even much higher doses of *gpc4* mRNA produced morphologically wild-type embryos [38]. Nonetheless, injecting embryos with 5 pg/nL of *gpc4* message enhanced the effects of injecting low (10 pg/nL) or high (60 pg/nL) levels of *wif1* message, as indicated by an increase in both the fraction of short-tailed embryos and the severity of the defects (Figure 4). Since the dose *gpc4* message we used has no effect on its own, these data indicate that Gpc4 enhances the activity of Wif1, consistent with the binding observed between human WIF1 and glypican HS sidechains [7]. We were not able to test the effects of removing *gpc4* on the *wif1*-overexpression phenotype because *gpc4* mutants already have strong axis defects, likely due to loss of a co-receptor-like activity in Wnt signaling [38].

### Interactions between Wif1 and glypicans require EGF-like domains

*D. melanogaster* Shf is stabilized in the extracellular space by glypicans [2]. For instance, the levels of endogenous Shf are reduced in clones lacking the glypican Dally, and are increased in cells overexpressing it (Figure S5A, S5B). These interactions require the presence of a normal ‘EGF-like’ domain, since *shf^2^* mutants that harbor a mutation in the third ‘EGF-like’ domain do not respond to changes in Dally levels (Figure S5C, S5D). Completely removing Shf's ‘EGF-like’ domains also greatly reduces its activity: *UAS-shf*Δ*EGF* was unable to fully rescue Hh activity in *shf* nulls [2]. Similarly, the ‘EGF-like’ domains of WIF1 have been shown to bind HS in vitro [7].

We therefore tested the activities of a *wif1* construct lacking the ‘EGF-like’ domains (*wif1*Δ*EGF*). To bypass the variability in transgene transcription frequently caused by different genomic insertion sites, we used *pVal-UAS-wif1* and *pVal-UAS-wif1*Δ*EGF* constructs that integrate into a single, pre-selected genomic location [74]. The absence of the ‘EGF-like’ domains is unlikely to alter the stability of the recombinant protein, since both the Wif1 and Wif1ΔEGF were secreted *in vitro* by *D. melanogaster* S2 cells at equal levels (Figure S6).

*pVal-UAS-wif1ΔEGF was substantially less effective at disrupting wing margin development than pVal-UAS-wif1: driving pVal-UAS-wif1ΔEGF expression with nub-Gal4 caused a much smaller reduction in the number of wing margin bristles (Figure 3I, 3K; quantified in Figure S3). Moreover, the effects of combining pVal-UAS-wif1ΔEGF with UAS-dlp on bristle numbers were additive, rather than synergistic (Figure 3L; quantified in Figure S3). Co-misexpression of pVal-UAS-wif1ΔEGF with UAS-dlp did not induce the synergistic wing margin scalloping that was observed after co-misexpression of pVal-UAS-wif1 and UAS-dlp (Figure 3J, 3L). In addition, Wif1ΔEGF did a much poorer job than full length Wif1 at increasing the accumulation of ex-Wg on the surfaces of wing disc cells expressing high levels of endogenous Dlp: in nub-Gal4, pVal-UAS-wif1ΔEGF discs, ex-Wg remained high along the prospective wing margin and low proximally (Figure 2N, 2O).*

In the context of zebrafish Wnt signaling, Wif1ΔEGF was also much less effective, and did not show comparable synergistic increases in its activity when with co-injected with *gpc4* (Figure 4). Therefore, the ‘EGF-like’ domains of Wif1 are important for interactions with glypicans in both *D. melanogaster* and zebrafish. However, it was recently reported that the ‘EGF-like’ domains also contribute to Wnt binding, providing an additional mechanism for the reduced activity of the Wif1ΔEGF constructs [7].

### The EGF-like domains are interchangeable between Wif1 and Shf

**Figure 5:**
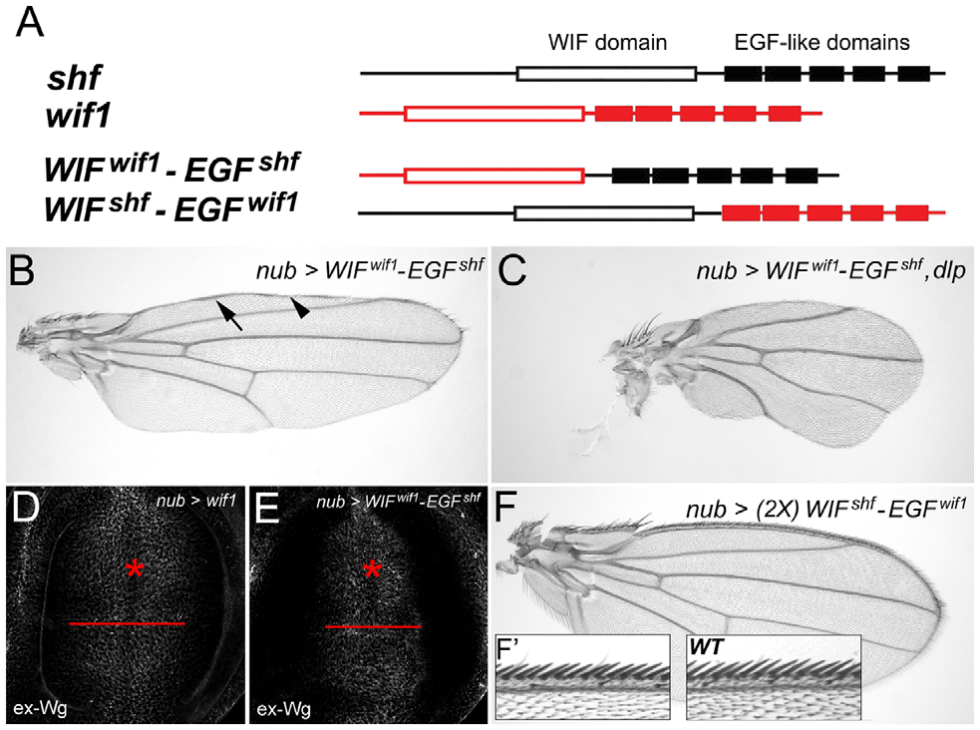
The EGF-like domains are interchangeable between Wif1 and Shf during Wif1-dependent Wg inhibition. (A) Domain compositions of Wif1, Shf and the two chimeric constructs. Open boxes show the ‘WIF’ domain, filled boxes the EGF-like domains. (B–F) *nub-Gal4*-driven misexpression of respective transgenes. (B) *UAS-WIF^wif1^-EGF^shf^* strongly reduces the density of anterior wing margin bristles and interrupts L1. Arrow and arrowheads denote L1 or lack of thereof, respectively. (C) Co-expression of *UAS-WIF^wif1^-EGF^shf^* and *UAS-dlp* almost completely eliminates wing margin bristles and L1, and reduces the size of the wing. (D, E) Expression of either *UAS-wif1* or *UAS-WIF^wif1^-EGF^shf^* similarly reduces ex-Wg levels on the surface of prospective margin cells (asterisks) and increases levels proximally. However, compared to *UAS-wif1*, *UAS-WIF^wif1^-EGF^shf^* expression does not increase ex-Wg as far proximally (compare red bars). (F, F′) Expression of two copies of *UAS-WIF^shf^-EGF^wif1^* does not alter wing shape or size, and has no measurable effect on margin bristles (anterior margin details in F′).

Unlike vertebrate WIF1, *D. melanogaster* Shf cannot inhibit Wg signaling [2], [3]. To investigate the domains responsible for this difference, we generated chimeric constructs in which we swapped the ‘WIF’ and ‘EGF-like’ domains between Wif1 and Shf (Figure 5A). WIF^Wif1^-EGF^Shf^ denotes a construct bearing zebrafish ‘WIF’ domain fused to the ‘EGF-like’ repeats of Shf (Figure 5). This construct affected Wg in a manner similar to that of full-length Wif1, causing wing margin defects in the adult wings, and redistributing ex-Wg in wing discs. All three *UAS-WIF^wif1^-EGF^shf^* transgenic lines tested caused adult wing defects comparable to that of our strongest *UAS-wif1* lines (Figure 5B), and also synergized with *UAS-dlp* (Figure 5C). Only two pieces of evidence indicate that the WIF^Wif1^-EGF^Shf^ chimera is not as potent as the full-length Wif1: 1) in wing discs *nub-Gal4*-driven expression of *UAS-WIF^wif11^-EGF^shf^* did not extend ex-Wg as far proximally as *UAS-wif1* (Figure 5D, 5E), and 2) *en-Gal4*-driven expression of *UAS-WIF^wif1^-EGF^shf^* yielded adult escapers, but *en-Gal4*-driven expression of *UAS-wif1* did not. Nonetheless, these data show that the ‘EGF-like’ domains of Wif1 and Shf are largely interchangeable during Wnt inhibition, suggesting that either can interact with HSPGs.

In contrast, expression of one or even two copies of the reciprocal chimera, *UAS-WIF^shf^-EGF^wif1^*, had no effect on Wg signaling (Figure 5F, 5F′). This indicates that the ‘WIF’ domain of Shf cannot interact with Wg strongly enough to inhibit signaling, even in the presence of the Wif1's ‘EGF-like’ domains. This also indicates that the ‘EGF-like’ domains of Wif1 cannot interact with Wg strongly enough to inhibit signaling, despite the presence of an orthologous ‘WIF’ domain. Thus, the specificity for Wnt inhibition resides in the WIF domain of Wif1.

We did, however, find sensitized contexts in which Shf weakly affected Wg signaling, albeit in an unexpected direction: co-expression of *UAS-shf* slightly improved the adult wing margin defects caused by expression of *UAS-wif1* or *UAS-Dfz2-GPI*, although not the wing margin defects caused by expression of *UAS-wg RNAi* (Figure S7 and data not shown). Thus, Shf weakly promotes Wg signaling in these contexts, the opposite of Wif1. Conversely, while expressing *UAS-wif1* with *nub-Gal4* yielded viable adults, *shf^2^* larvae expressing *UAS-wif1* did not survive to adulthood; this suggests that endogenous Shf can counteract the otherwise lethal Wnt-inhibitory effects of Wif1. We will present possible mechanisms for these effects in the Discussion.

### The WIF domain of Wif1 can regulate Hh signaling

**Figure 6:**
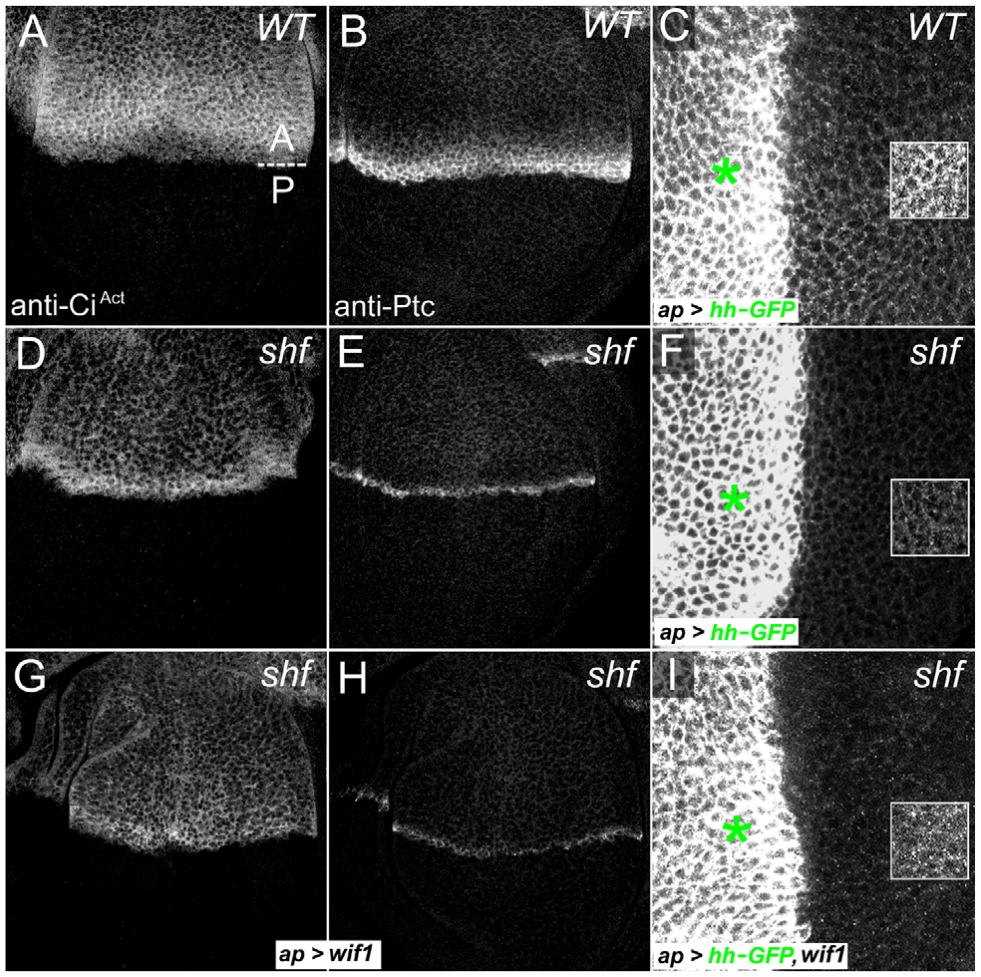
Wif1 affects Hh activity in *shf* discs. (A–C) Wild-type wing discs, showing the regions with high levels (red bars) of Ci^Act^ (A) or Ptc (B), or showing the ventral accumulation of Hh-GFP in the posterior compartment after dorsal, *ap-Gal4*-driven expression of *UAS-hh-GFP* (C). (D–F) *shf*/*Y* wing discs show reductions in the width of domains expressing high levels of Ci^Act^ (D) or Ptc (E), and also show reduced ventral accumulation of Hh-GFP in the posterior compartment after dorsal *ap-Gal4*-driven expression of *UAS-hh-GFP* (F). (G–I) *shf*/*Y* wing discs with *ap-Gal4*-driven expression of *UAS-wif1* have a broader domain of less intense anti-Ci^Act^ staining (G) and lower levels of anti-Ptc staining (H), but improve the ventral accumulation of Hh-GFP in the posterior compartment after *ap-Gal4*-driven expression of *UAS-hh-GFP* (I). Ventral Hh-GFP was more punctuate than in wild type discs (compare to C). To make it easier to see the differences in ventral Hh-GFP accumulation, levels were increased equally in the boxed regions in C, F, and I. Hh-GFP levels are quantified in Figure S9.

**Figure 7:**
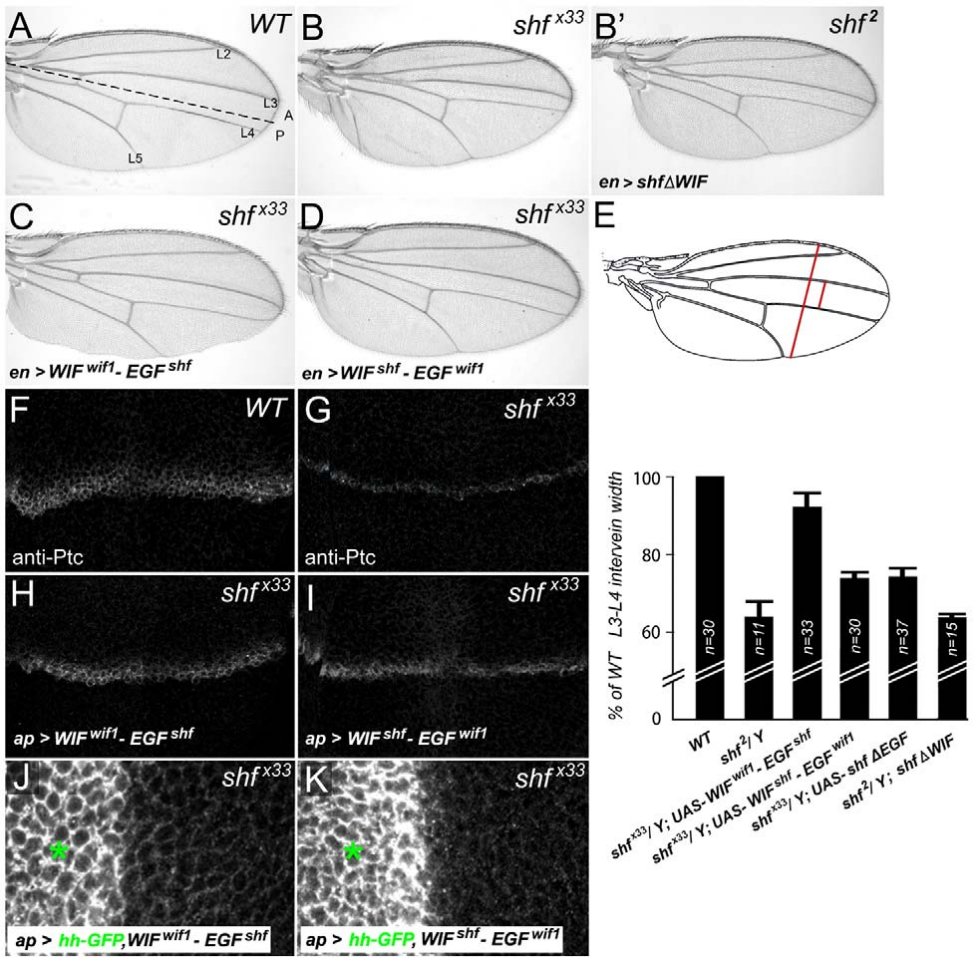
Vertebrate WIF domain regulates long-range Hh signaling. (A) Wild type wing showing the positions of the first through fifth longitudinal veins (L1–L5) and the position of the A/P compartment boundary (dashed line). (B) Reduced L3–L4 spacing in *shf* mutant wing (B) is not improved upon expression of *UAS-shf*Δ*WIF* (B′). (C, D) Posterior, *en-Gal4*-driven expression of *UAS-WIF^wif1^-EGF^shf^* (C) or *UAS-WIF^shf^-EGF^wif1^* (D) in *shf^x33^*/*Y* wings. *UAS-WIF^wif1^-EGF^shf^* strongly improved and *UAS-WIF^shf^-EGF^wif1^* weakly improved L3–L4 spacing. (E) Comparison of L3–L4 spacing in wild type, *shf^2^*, and *shf^x33^/Y* with *en-Gal4*-driven *UAS-construct* expression. To compensate for differences in overall wing size, we normalized the L3–L4 distance to the distance between the anterior and posterior margins (red bars). In all but one case we presented the experimental normalized L3–L4 distances as percentages of the wild type normalized distances. However, since expression of *UAS-WIF^wif1^-EGF^shf^* reduced the size of the posterior compartment, and thus the distance between the anterior and posterior margins, we compared the normalized L3–L4 distance in *shf^x33^ UAS-WIF^wif1^-EGF^shf^ wings* to the normalized L3–L4 distance in non-*shf UAS-WIF^wif1^-EGF^shf^* siblings (*n = 31*). Bars denote standard deviation. Two-tailed Student's t test showed no significant differences between *UAS-WIF^shf^-EGF^wif1^* and *UAS-shfΔEGF*, or between *shf^2^* and *shf^2^*, *UAS-shfΔWIF*. Differences between the other conditions were significant (*p*<0.0001). (F–I) Anti-Ptc staining in wild type (F), *shf^x33^*/*Y* (G), and *shf^x33^*/*Y* wing discs with *ap-Gal4*-driven expression of *UAS-WIF^wif1^-EGF^shf^* (H) or *UAS-WIF^shf^-EGF^wif1^* (I). The improvement in the width of Ptc expression in *shf^x33^* discs was stronger after expression of *UAS-WIF^wif1^-EGF^shf^* than *UAS-WIF^shf^-EGF^wif1^*. (J, K) *shf^x33^*/*Y* wing discs with *ap-Gal4*-driven expression of *UAS-hh-GFP* and *UAS-WIF^wif1^-EGF^shf^* (J) or *UAS-hh-GFP* and *UAS-WIF^shf^-EGF^wif1^* (K). *UAS-WIF^wif1^-EGF^shf^* strongly improved the ventral accumulation of Hh-GFP, while the improvement with *UAS-WIF^shf^-EGF^wif1^* was more modest (quantifications in Figure S9).

Previous results suggested that neither human WIF1 nor its fish homolog could promote strong Hh signaling in *D. melanogaster* [2], [3]. Through a more careful examination, however, we found that Wif1 can alter Hh signaling, albeit weakly. In the wing disc, Hh is produced by the cells of posterior (P) compartment and signals to adjacent cells of the anterior (A) compartment [75]. Antibodies to the activated form of the Gli-family transcription factor Cubitus interruptus (Ci^Act^, NCBI Gene ID: 43767), and to the Hh receptor Patched (Ptc, NCBI Gene ID: 35851), measure low- and high-threshold Hh responses, respectively [76], [77]. Signaling can also be measured in the adult wing, since the anterior-posterior distance between the longitudinal wing veins L3 and L4 is regulated via transcription of *knot* (also known as *collier*), a high-threshold Hh target [76], [78]–[82]. In *shf* adult wings the spacing between L3 and L4 is greatly reduced, and in wing discs the normally broader domains of anti-Ci^Act^ and anti-Ptc staining regress to thin stripes [2], [3] (Figure 6A, 6B, 6D, 6E and Figure 7A, 7B). *shf* is also required for the extracellular accumulation and movement of Hh: when GFP-tagged Hh (Hh-GFP) is expressed in dorsal cells using *ap-Gal4*, it accumulates in adjacent ventral cells (Figure 6C), but in *shf* mutants the ventral accumulation of dorsally-expressed Hh-GFP is largely lost (Figure 6F). Because Shf is quite diffusible, all of these defects can be rescued by expression of *UAS-shf* from any domain in the wing disc [2], [3].

We were unable to test the effects of Wif1 on L3–L4 spacing in the adult *shf* mutant wings, since expressing *UAS-wif1* in *shf* mutants using any of several drivers, including *nub-Gal4*, caused pupal lethality (see below). Nonetheless, expression of *UAS-wif1* using *ap-Gal4* significantly increased the ventral accumulation of dorsally expressed Hh-GFP in wing discs mutant for the null allele of *shf* (*shf^x33^*) (Figure 6I; quantified in Figure S7). This accumulation was not normal, however: it appeared more punctate than the ventral Hh-GFP accumulation observed in control discs. This abnormal Hh accumulation may account for its effects on signaling. Instead of the narrow domain of intense anti-Ci^Act^ staining normally observed anterior the compartment boundary of *shf* discs, the staining now appeared less intense, broader and more uniform after *wif1* expression (Figure 6G). In some *shf^x33^* discs, *UAS-wif1* expression also appeared to reduce the intensity of the high-threshold Hh target Ptc, although the width of the anti-Ptc staining was not affected (data not shown).

To make sure that the change in anti-Ci^Act^ staining induced by Wif1 was not an indirect effect caused by reduced Wg signaling, we reduced Wg signaling in *shf* discs using *UAS-Dfz2-GPI*. This did not alter anti-Ci^Act^ staining (Figure S8), despite the strong effects of *UAS-Dfz2-GPI* on Wg signaling and wing margin development (Figure S2C). This is consistent with previous findings that changes in Wg signaling do not obviously affect anti-Ci^Act^ staining or Hh signaling in the wing [2], [3], [45].

We next used the chimeric constructs described above to investigate the protein domains responsible for the different Hh signaling activities of Wif1 and Shf, again measuring their ability to rescue the *shf* mutant phenotypes. Expression of *UAS-WIF^wif1^-EGF^shf^* in *shf^x33^* mutants restored the wing vein phenotype to nearly wild-type (Figure 7A–7C, 7E) and greatly increased both the width of the region expressing the high-threshold Hh target Ptc and the ventral accumulation of dorsally-expressed Hh-GFP (Figure 7F–7H, 7J; quantified in Figure S9). Since expression of the Shf's ‘EGF-like’ domains on their own (ShfΔWIF) cannot improve *shf^2^* mutant defects [83] (Figure 7B′, 7E), these results reveal that the zebrafish ‘WIF’ domain can be highly active in Hh signaling, as long as it is coupled to the EGF-like domains of *D. melanogaster* Shf.

In contrast, the effects of the *UAS-WIF^shf^-EGF^wif1^* chimera on L3–L4 spacing, anti-Ptc staining and Hh-GFP movement in *shf* wings and discs were much more modest (Figure 7A, 7B, 7D, 7E, 7G, 7I, 7K; quantified in Figure S9), which was very similar to the weak effects of Shf's WIF domain alone (*UAS-shf*Δ*EGF*, Figure 7E) (A.A. and S.S.B submitted). This indicates that Wif1's ‘EGF-like’ domains contribute little to the regulation of Hh signaling.

## Discussion

### Wif1's role in regulating Wnt-glypican interactions

We have shown that, in the model system provided by the developing *D. melanogaster* wing, the inhibition of Wg signaling caused by Wif1 expression is accompanied by the accumulation of extracellular Wg on the surfaces of cells that have high concentrations of the glypican Dlp. Dlp was necessary and sufficient for much of this effect: the accumulation of extracellular Wg is largely eliminated by reducing Dlp levels and increased by increasing Dlp levels; genetic interactions also suggest a partly redundant role for the other *D. melanogaster* glypican, Dally (see below). WIF1 binds both Wnts, largely through its ‘WIF’ domain [1], and the HS sidechains of glypicans, largely through its ‘EGF-like’ domains [7]. Thus, WIF1 likely reinforces Wnt-glypican binding by forming a complex with both, similar to the role proposed for the *D. melanogaster* Wif1 homolog Shf, which binds Hh and glypicans and stabilizes Hh on cell surfaces [2], [3].

But while the stabilization of Hh-glypican interactions by Shf is accompanied by an increase in Hh signaling, our evidence shows that the increase in Wnt-glypican binding caused by WIF1 does not increase signaling. In fact, the glypican interactions strongly potentiate Wif1's Wnt-inhibiting activity: the *D melanogaster* glypicans Dlp and Dally increase the effectiveness of Wif1 in developing wings, while the zebrafish glypican Glp4 (also known as Knypek) increases the effectiveness of Wif1 in zebrafish embryos. Previous studies suggest that human WIF1 expression reduces the receptor-mediated endocytosis of Wg in the wing disc [3], so the complex of Wif1 and glypicans apparently either sterically blocks Wg's ability to bind receptors, or sequesters Wif1-bound Wg into an extracellular, glypican-rich domain with less access to the receptors. HSGP interactions are similarly thought to potentiate the modulatory effects of BMP-binding proteins, such as Noggin [84], Chordin [85] and Crossveinless-2/BMPER (Cv-2) [86]–[88].

In vitro evidence suggests that WIF1 binds the HS sidechains of HSPGs through its ‘EGF-like’ domain [7]. This is consistent with our data on the *D. melanogaster* Wif1 ortholog Shf; Shf accumulation in wing discs is sensitive to glypican and HS levels, but that sensitivity is lost after a missense mutation in Shf's third ‘EGF-like’ domain [2]. We show that the loss of Wif1's ‘EGF-like’ domains, and thus HS binding, greatly reduces Wif1's ability to increase Wg accumulation on glypican-expressing cells, consistent with direct role for a Wg-Wif1-glypican complex.

We also found that loss of the ‘EGF-like’ domains greatly reduces Wif1's effectiveness in both *D. melanogaster* and zebrafish embryos, and that removing the ‘EGF-like’ domains reduced the synergism between Wif1 and Dlp in wing discs, and between Wif1 and the zebrafish glypican Gpc4 in zebrafish embryos. A recent study also found that removing the ‘EGF-like’ domains reduced human WIF1's effectiveness in an in vitro assay; however, the ‘EGF-like’ domains were also reported to reinforce the binding of Wnts to the WIF domain [7], so it is uncertain whether the reduction of Wif1 activity can be attributed wholly to the loss of HS binding. Interestingly, in other published assays loss of the ‘EGF-like’ domains did not obviously reduce WIF1 activity [1]. This suggests that the HS interactions are not important for the inhibition of Wnt signaling in all contexts. This parallels the activity of the BMP-binding and glypican-binding protein Cv-2, since removal of its HSGP-binding domain greatly reduces its ability to inhibit BMP signaling in some assays, but not others [86]–[89].

### The biphasic effects of the glypicans on Wnt signaling

One consequence of the interaction between Wif1 and glypicans is that it alters the effects of glypicans on signaling. Increasing the levels of Dlp or Dally in the presence of WIF1 synergistically decreases Wg signaling, and reducing the levels of endogenous Dlp or Dally increases Wg signaling. The latter result is particularly telling, because in the absence of WIF1 removing endogenous Dally reduces Wg signaling in the wing [32], [33], [36]. Thus, WIF1 can change a glypican's role from the stimulation to the inhibition of Wnt signaling. We observed similar genetic interactions in zebrafish embryos between WIF1 and zebrafish Gpc4.

This underscores the complexity of the role glypicans play in regulating signaling. In some settings the effects of the glypicans on Wnt are known to be biphasic. While endogenous Dally weakly stimulates Wg signaling in the wing disc [32], [33], [36], we and others have shown that overexpression of Dally can inhibit Wg-dependent signaling in the embryo and during wing margin development [90], [91]. Endogenous Dlp inhibits signaling close to the distal, *wg*-expressing cells of the wing margin, but stimulates signaling in proximal cells distant from the wing margin [30]–[35], [39]. One explanation proposed for these different effects is spatial: Dlp may sequester excess Wg from its receptors distally, near the site of Wg secretion, but increase the movement of Wg from distal to proximal cells, increasing the amount of Wg that is available for proximal signaling. But Dlp can also both stimulate and inhibit Wg signaling in vitro, where all cells are likely to have access to the Wg in the culture medium [30], [39]. Low levels of Dlp stimulate Wg signaling, while high levels inhibit; the biphasic effects of Dlp are also influenced by the levels of Wg and the DFz2 receptor, favoring stimulation when the levels of Wg are low and the levels of DFz2 are high, but favoring inhibition when the levels of Wg are high and the levels of DFz2 are low.

Vertebrate glypicans such as Gpc4 can be similarly biphasic. Removing Gpc4 inhibits non-canonical Wnt signaling in zebrafish embryos, indicating a positive role in signaling, and while overexpression of Gpc4 does not inhibit signaling on its own, at high levels it makes *wnt11* mRNA less effective at rescuing zygotic *wnt11* mutants [38]. Other vertebrate glypicans have also been reported to inhibit Wnt activity in various contexts [92].

A mathematical model using a different cell surface ligand-binding protein, the BMP-binding protein Cv-2, provides one way of explaining such biphasic effects [87]. If a ligand-binding protein can exchange ligand directly with the receptor, it may either provide more ligand for the receptor or sequester ligand from the receptor. The model suggests that, within certain ranges of binding constants, the signaling outcome will be positive with lower concentrations of the ligand-binding protein, and negative with higher concentrations.

The ability of glypicans to interact with other Wnt-binding molecules provides another way of altering the biphasic activity of glypicans. Since Wif1 increases the amount of Wnt binding to the glypican, this should increase the glypican's effective concentration, biasing its biphasic activity towards inhibition. Thus, the presence or absence of proteins that bind both glypicans and Wnts may provide an explanation for some of context-specific activities of vertebrate glypicans.

### Pathway specificity of Wif1 and Shifted

The ‘WIF’ domain of WIF1 does not bind HS sidechains, but is sufficient for Wnt binding; the ‘EGF-like’ domains show only weak binding to Wnts on their own, but appear to strengthen Wnt binding to the ‘WIF’ domain [1], [7]. But while the *D. melanogaster* WIF1 homolog Shf contains both ‘WIF’ and ‘EGF-like’ domains, it does not inhibit Wg signaling; instead, it increases the levels or range of Hh signaling [2], [3]. We found that a construct containing Shf's ‘WIF’ domain and the zebrafish Wif1's ‘EGF-like’ domains also cannot inhibit Wnt signaling, while the reciprocal construct with Wif1's ‘WIF’ domain and Shf's ‘EGF-like’ domain can. Similar results have been obtained with constructs made from Shifted and human WIF1 (I. Guerrero, personal communication). Thus, the ability to inhibit Wg activity, and likely to bind significant levels of Wg, resides in the different ‘WIF’ domains of Wif1 and Shf.

Surprisingly, Shf did show a weak ability to improve Wg signaling in sensitized backgrounds expressing either Wif1 or the dominant negative DFz2-GPI construct. While we have never detected any obvious effect of Shf on ex-Wg levels, it may weakly interact with Wg in a manner that reduces the levels bound to Wif1 or DFz2-GPI and increases the levels available for the Wg receptors. Consistent with this interpretation, *UAS-shf* did not alleviate margin defects caused by expression of *UAS-wg RNAi*, even though *UAS-Dfz2-GPI* and *UAS-wg RNAi* show a very comparable impact on Wg activity. Alternatively, Shf's effect on Wnt signaling might be due to interactions with the Wnt4 or Wnt6 expressed along the wing margin, which may have redundant roles in wing margin development [42] that are only obvious in a sensitized background. Indirect effects via Hh signaling are unlikely, as Shf overexpression does not further increase Hh signaling [2], [3].

The situation with Hh signaling is more complex. First, vertebrate WIF1's are not known to regulate vertebrate Hh signaling, but we found that zebrafish Wif1 can weakly affect the reduced movement or accumulation of Hh normally observed in *shf* mutant wing discs. The Hh-GFP accumulation is abnormal, however, appearing more punctuate than in normal wing discs, perhaps accounting for its ability to reduce the expression of Hh targets.

Placing WIF domain of zebrafish Wif1 in the context of Shf's ‘EGF-like’ domains in a chimeric WIF^Wif1−^EGF^Shf^ construct almost fully rescues loss of *shf* function, something not observed after expression of the Shf ‘EGF-like’ domains alone. Together, these data suggest that the ‘WIF’ domains of both Shf and zebrafish Wif1 are capable of interacting with Hh. Like Wnts, Hh is palmitoylated [93], and it has been suggested that these palmitates might bind a hydrophobic pocket found in the WIF domain [94], [95], although this has been recently questioned [7]. The activity of ‘WIF’ domains in Hh signaling may also vary between different vertebrates, since unlike the WIF^Wif1^–EGF^Shf^ construct made using zebrafish ‘WIF’ domains, a similar construct made using the ‘WIF’ domain from human WIF1 does not rescue loss of *shf* function (I. Guerrero, personal communication).

The Shf ‘EGF-like’ domains are necessary to confer a Shf-like level of Hh-promoting activity to the ‘WIF’ domains of zebrafish Wif1. The Hh-promoting activity of Wif1's ‘WIF’ domain is increased by placing it in the context of Shf's ‘EGF-like’ domains, and the low Hh-promoting activity of Shf's ‘WIF’ domain is not changed by placing it in the context of Wif1's ‘EGF-like’ domains. It is unlikely that the ‘EGF-like’ domains of Shf and Wif1 differ significantly in their HSPG-binding activities, since Wif1 and WIF^Wif1^-EGF^Shf^ differ only slightly in their ability to inhibit Wnt signaling and interact genetically with Dlp. We therefore favor the alternative hypothesis that Shf's ‘EGF-like’ domains contribute to Hh signaling through a mechanism independent of glypican binding. While the Shf ‘EGF-like’ domains alone (ShfΔWIF) cannot increase Hh signaling, we have found that they can increase the levels of extracellular Hh (A.A. and S.S.B., submitted), suggesting that they contribute to Hh binding, much as the ‘EGF-like’ domains of WIF1 do to Wnt binding [7].

Since Wif1 can alter Hh distribution and, more weakly, signaling in *D. melanogaster*, an important question is whether it can also do so in vertebrates. Because of its strong effects on Wnt signaling, vertebrate WIF1 family proteins have rarely been assayed for their effects on other pathways, so a weak modulation of one of the vertebrate Hhs remains a possibility.

## Materials and Methods

### Ethics statement

Animals were handled in accordance with guidelines set forth by NIH and IACUC. Our animal use protocols were approved by the University of Wisconsin and Tufts University Institutional Animal Care and Use Committees

### Molecular constructs and transgenic flies

*pUAS-wif1* and *pVal-UAS-wif1* were generated by PCR from full-length *wif1* template [2]. *pVal-wif1ΔEGF* terminates at R^177^; both the pVal inserted constructs also contain a C-terminal V5 epitope. Constructs were expressed in S2 cells as described [96], and checked for expression on Western blots using standard procedures. The chimeric Shf/Wif1 coding sequences were generated using PCR and were spliced between the end of the ‘WIF’ domain and the beginning of the first ‘EGF-like’ domains with junctions at E^282^:Q^180^ (WIF^Shf^-EGF^Wif1^) and T^174^:C^261^ (WIF^zWIF1^-EGF^Shf^). WIF^Shf^-EGF^Wif1^ also contains a V5 epitope N-terminal to the WIF domain. The identically tagged full-length Shf localized and functioned like endogenous Shf protein (A.A., S.H., S.S.B; unpublished). Most transgenes were subcloned into pUAST, but for the comparison between Wif1 and Wif1ΔEGF they were subcloned into pValium1 and intergrated into identical *attP2* genomic sites [74]. Construct DNA was injected into *D. melanogaster* embryos by Injection Services, Inc. (Sudbury, MA).

### Zebrafish experiments

*gpc4* open reading frame was generously provided by L. Solnica-Krezel. mRNA was prepared *in vitro* from linearized plasmids using mMessage mMachine Kit (Ambion), purified and kept at −80 C° in frozen aliquots. Before injection, freshly thawed mRNA was diluted to stock concentrations depicted in Figure 4 in 0.1 M KCl containing small amounts of phenol-red for tracing purposes. Approximately 2 nL of injection mixture was injected into one cell stage embryos as described [97]. Injected embryos were allowed to recover at 28.5°C in embryo medium and 28–30 hour old embryos were evaluated for defects in posterior development. To evaluate canonical Wnt signaling, homozygous *Tg(TOP:dGFP)* fish were crossed with wild-type and their F1 progeny were injected with the chosen mRNAs as described above. To measure GFP fluorescence 30 hour old embryos were mounted in low melting point agarose on coverslips and photographed using an EM-CCD camera (Photometrix) under constant exposure settings. Calculations of GFP intensity were performed in ImageJ and presented in arbitrary units and compared to the levels of the wild-type controls that were set at 100% after normalization.

### *D. melanogaster* strains and genetics

Flies were maintained at 25°C. Mutant analyses used *shf^x33^* [2], *shf^2^* [98]; *dally^80^* and *dlp^A187^* [26]. Mutant clones were generated using FRT-mediated mitotic recombination [99]. UAS-transgenes were expressed using *ap-Gal4*, *dpp-Gal4*, *en-Gal4*, or *nub-Gal4* (Bloomington, IN) [100], or in Flpout-Gal4 clones using *y,w, hs-Flp; Act>y^+^>Gal4 UAS-GFP* and a 60 minute 37°C heat shock at three days after egg laying. In addition to the UAS lines generated above, we used: *UAS-dally* [101], *UAS-dlp* [34], *UAS-wif1*, *UAS-shf*, *UAS-shf*Δ*EGF*, *UAS-shf*Δ*WIF* [2], *UAS-arm^S10^* [53], *UAS-Dfz2-GPI* [102], *UAS-hh-GFP* [103], *UAS-dlp RNAi* (Vienna *Drosophila* RNAi Center 10299) and *UAS-wg RNAi* (Transgenic RNAi Project JF01480).

### Immunohistochemistry

Late third instar discs were dissected in ice cold PBS and fixed in EM-grade 4% formaldehyde/PBS for 30 minutes at 4°C, rinsed in PBS containing 0.2% Triton X-100, and incubated with primary antibodies overnight at 4°C. Primary antibodies and their working dilutions were: mouse anti-Wg (1∶50; Development Studies Hybridoma Bank [DSHB], Iowa City) [104], rabbit anti-GFP (1∶200, MBL International), mouse anti-GFP (1∶200, Chemicon), mouse anti-V5 (1∶200, Invitrogen), rabbit anti-V5 (1∶200, Bethyl), mouse ant-Dlp (1∶200; DSHB) [105], guinea pig anti-Sens (1∶200) [47], rat anti-Ci^Act^ (1∶20) [77], and/or mouse anti-Ptc (1∶200; DSHB) [106], followed by incubation with fluorescent secondary antibodies (Jackson Immunoresearch). Images were acquired using laser scanning confocal microscopy.

Extracellular Wg was detected by incubating discs with anti-Wg (1∶3) for 30–45 minutes at 4°C in Shields and Sang M3 culture medium, briefly rinsing in PBS, fixing for 30 min in 4% formaldehyde/PBS and staining with secondary antisera in PBS [50]. In some cases this was followed by staining for additional antigens as described above.

## Supporting Information

Figure S1 Reductions in Wg signaling disrupt wing margin development. Comparison of wild type adult wing (A) featuring dense array of anterior sensory bristles along longitudinal vein 1 (L1, arrow), and wing resulting from *nub-Gal4*-driven expression of *UAS-wg RNAi* in the prospective wing blade (B). Knockdown of *wg* expression causes loss of wing margin bristles, loss of L1 (arrow) and scalloping of the wing margin. Anterior is up and distal is to the right.
(PDF)

Figure S2 Expressing DFz2-GPI with Dlp or Wif1. (A) Wild-type wing with normal wing margin and margin bristles. (B) *nub-Gal4*, *UAS-wif1* wing almost completely lacks anterior bristles and exhibits moderate notching of the wing margin. (C) *nub-Gal4*, *UAS-Dfz2-GPI* wings lack all margin bristles, and shows extreme notching of the wing margin and reduced wing size. (D). *nub-Gal4*, *UAS-Dfz2-GPI*, *UAS-wif1* wings showed further reduction of wing size than with *nub-Gal4 UAS-Dfz2-GFP* alone, indicating that *nub-Gal4*, *UAS-Dfz2-GFP* are responsive to further reductions in Wnt/Wg signaling. (E) *nub-Gal4*, *UAS-Dfz2-GPI UAS-wif1* showed similar effects as *nub-Gal4 UAS-Dfz2-GFP* alone, showing that expression of Dlp does not enhance of the effects of DFz2-GPI expression.
(PDF)

Figure S3 Effects of Wif1 variants and Dlp on stout bristle differentiation. Expression of Dlp together with moderately driven *pVal-wif1* or *pVal-wif1ΔEGF* genomic insertions produced additive reductions in bristle number. Due to slight variability in wing size, stout bristle number was normalized to the length of the sampling area defined in Arbitrary Units (A.U.) The sampling area began at the distal point where L2 intercepts the wing margin (see Figure 7A for L positions) and extended proximally along the anterior margin. The normalized stout bristle numbers were not different between the *nub-gal4*, *pVal-UAS-wif1* and *nub-gal4*, *pVal-UAS-wif1ΔEGF*, *UAS-dlp* conditions. All other differences were statistically significant as determined by the two-tailed Mann-Whitney U Test (*p<0.015*).
(PDF)

Figure S4 Wif1 expression in zebrafish embryos leads to decreased expression of a β-catenin-regulated reporter. (A, A′) DIC (A) and fluorescence (A′) images of embryos carrying the *Tg*(*TOP:dGFP*) reporter, showing GFP expression in the dorsal midbrain (arrow), anterior to the midbrain-hindbrain boundary. (B) Injection of 40 pg/nL of *wif1* mRNA reduces GFP expression (arrow). (C) Injection of 5 pg/nL of *kny* mRNA does not change GFP expression. (D) GFP intensities were measured in living embryos and their statistically significant differences were verified by two-tailed Mann-Whitney U test. Statistically significant differences in GFP intensity were measured. No differences of statistical significance from were observed between wild-type and *kny*-injected embryos.
(PDF)

Figure S5 Shf interactions with the *D. melanogaster* glypican Dally require normal ‘EGF-like’ domains. (A, B) Changes in anti-Shf staining (red) in wild type discs (WT) containing *dally* mutant clones (A) or overexpressing *dally* (B). Anti-Shf staining is reduced (asterisk) in a *dally* mutant clone (marked by the absence of GFP in green) in the anterior compartment (A). Dorsal, *ap-Gal4*-driven expression of *UAS-dally* (marked using *UAS-GFP*, green) stabilizes Shf (B, asterisk). (C, D) In *shf^2^*, discs, anti-Shf staining is not reduced in an anterior *dally* mutant clone (C, asterisk), or increased by dorsal, *ap-gal4* driven *UAS-dally* expression (D, asterisk). Shf^2^ protein contains a missense mutation in Shf's third “EGF-like’ domain.
(PDF)

Figure S6 Full length and EGF-depleted Wif1 are secreted by *Drosophila* S2 cells. Constructs were tagged with V5 epitope at their C-termini (Materials and Methods). Respective *pVal-UAS-wif1-V5* and *pVal-UAS-wif1ΔEGF-V5* were co-transfected with *pAW-Gal4*. Supernatants were harvested at day 5 post-transfection. The low molecular weight band represents Wif1ΔEGF (lane A), which is fully stable since it is secreted at levels virtually equal to the levels of the full length Wif1 (lane B).
(PDF)

Figure S7 Shf partially alleviates Wg signaling defects in *UAS-wif1* or *UAS-Dfz2-GPI* expressing wings. (A, B) *UAS-shf* reduces notching defects in *UAS-wif1* expressing wings, restoring L1 and some anterior bristles. (C, D) *UAS-shf* increases wing growth in *UAS-Dfz2-GPI*-expressing wings. Wings expressing *UAS-shf* alone are indistinguishable from wild-type [2], [3].
(PDF)

Figure S8 The effects of Wg signaling on Ci^Act^ accumulation in *shf* mutants. (A) Strong inhibition of Wg signaling in a *shf^x33^*/*Y* wing disc, using *nub-gal4*- driven *UAS-Dfz2-GPI*, did not alter the width of the domain with strong accumulation of Ci^Act^. Compare to anti-Ci^Act^ staining in *shf* mutants in Figure 6D. (B) *nub-gal4*-driven expression of *UAS-wif1* in a *shf^x33^*/*Y* wing disc caused a broader, less intense domain of Ci^Act^.
(PDF)

Figure S9 Effects of Wif1 and chimeric Wif1:Shf proteins on Hh-GFP levels. Constructs were expressed in *shf* nulls (*shf^x33^*). The image in the upper panel shows areas sampled for the measurements, and the chart shows the GFP intensities in arbitrary units. Values obtained for the Posterior-Ventral quadrant (P–V) were normalized, and expressed as the percentage of signal intensity compared to the Anterior Ventral quadrant (A–V) where the Hh-GFP signal is at background levels. Graph shows averaged values and error bars show standard deviation. Differences of statistical significance were determined using the two-tailed Mann-Whitney U Test. In *shf* discs, Hh-GFP diffusion into the ventral compartment was significantly enhanced (asterisks) by Wif1 (*p = 0.023*) or WIF^Wif1^-EGF^Shf^ (*p<0.001*), but not significantly enhanced by WIF^Shf^-EGF^Wif1^ (*p = 0.153*).
(PDF)
