# Supplementary material for: The Role of Glypicans in Wnt Inhibitory Factor-1 Activity and the Structural Basis of Wif1's Effects on Wnt and Hedgehog Signaling
Source: PLoS Genet. 2012 Feb 23;8(2):e1002503. doi: 10.1371/journal.pgen.1002503 (PMC3285576; doi:10.1371/journal.pgen.1002503)
Supplement: Figure S1 — Reductions in Wg signaling disrupt wing margin development. Comparison of wild type adult wing (A) featuring dense array of anterior sensory bristles along longitudinal vein 1 (L1, arrow), and wing resulting from nub-Gal4-driven expression of UAS-wg RNAi in the prospective wing blade (B). Knockdown of wg expression causes loss of wing margin bristles, loss of L1 (arrow) and scalloping of the wing margin. Anterior is up and distal is to the right. (PDF) [file pgen.1002503.s001.pdf]

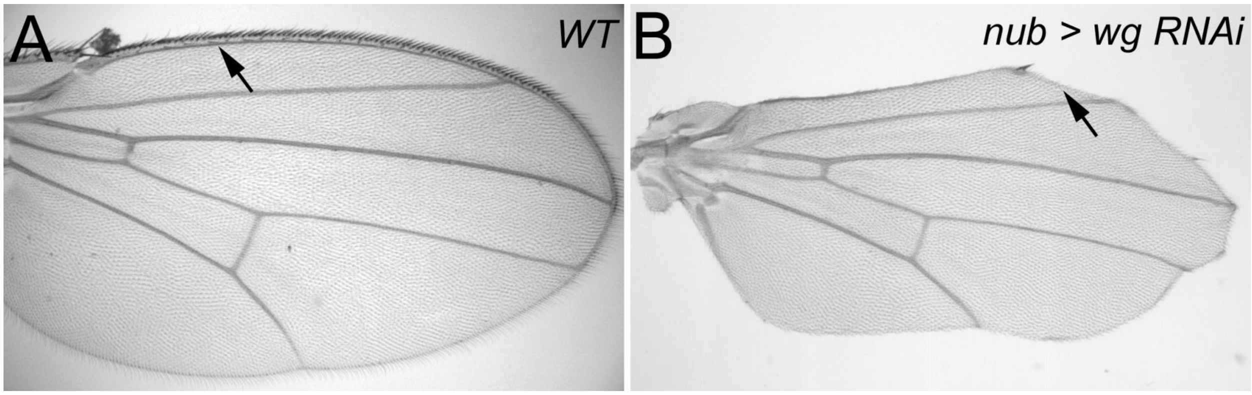


**Figure S1. Reductions in Wg signaling disrupt wing margin development**

Comparison of wild type adult wing **(A)** featuring dense array of anterior sensory bristles along longitudinal vein 1 (L1, arrow), and wing resulting from *nub-Gal4*-driven expression of *UAS-wg RNAi* in the prospective wing blade **(B)**. Knockdown of *wg* expression causes loss of wing margin bristles, loss of L1 (arrow) and scalloping of the wing margin. Anterior is up and distal is to the right.
