# Supplementary material for: The Role of Glypicans in Wnt Inhibitory Factor-1 Activity and the Structural Basis of Wif1's Effects on Wnt and Hedgehog Signaling
Source: PLoS Genet. 2012 Feb 23;8(2):e1002503. doi: 10.1371/journal.pgen.1002503 (PMC3285576; doi:10.1371/journal.pgen.1002503)
Supplement: Figure S2 — Expressing DFz2-GPI with Dlp or Wif1. (A) Wild-type wing with normal wing margin and margin bristles. (B) nub-Gal4, UAS-wif1 wing almost completely lacks anterior bristles and exhibits moderate notching of the wing margin. (C) nub-Gal4, UAS-Dfz2-GPI wings lack all margin bristles, and shows extreme notching of the wing margin and reduced wing size. (D). nub-Gal4, UAS-Dfz2-GPI, UAS-wif1 wings showed further reduction of wing size than with nub-Gal4 UAS-Dfz2-GFP alone, indicating that nub-Gal4, UAS-Dfz2-GFP are responsive to further reductions in Wnt/Wg signaling. (E) nub-Gal4, UAS-Dfz2-GPI UAS-wif1 showed similar effects as nub-Gal4 UAS-Dfz2-GFP alone, showing that expression of Dlp does not enhance of the effects of DFz2-GPI expression. (PDF) [file pgen.1002503.s002.pdf]

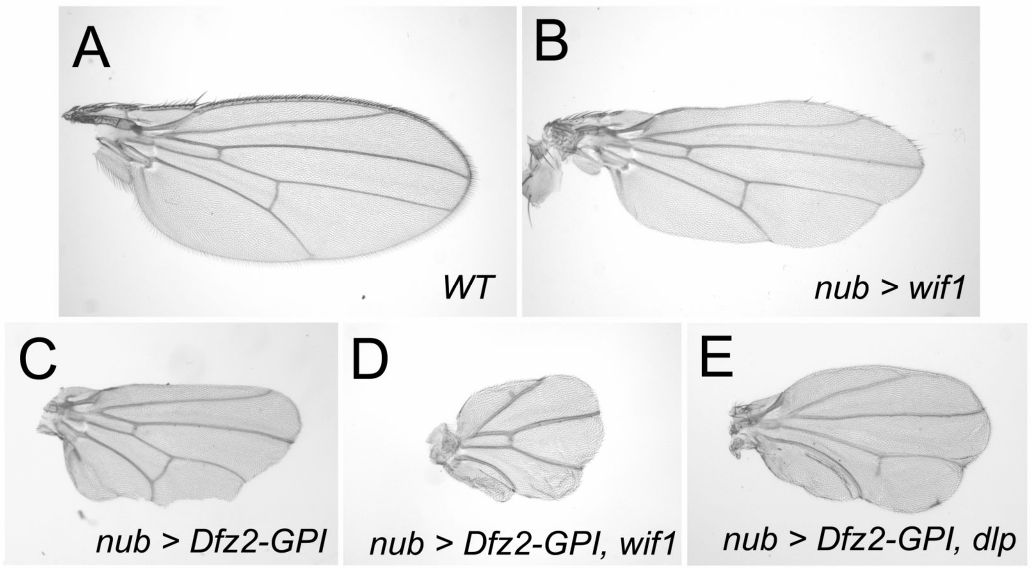

Figure S2. Expressing DFz2-GPI with Dlp or Wif1

(A) Wild-type wing with normal wing margin and margin bristles. **(B)** *nub-Gal4, UAS-wif1* wing almost completely lacks anterior bristles and exhibits moderate notching of the wing margin. **(C)** *nub-Gal4, UAS-Dfz2-GPI* wings lack all margin bristles, and shows extreme notching of the wing margin and reduced wing size. **(D)**. *nub-Gal4, UAS-Dfz2-GPI, UAS-wif1* wings showed further reduction of wing size than with *nub-Gal4 UAS-Dfz2-GFP* alone, indicating that *nub-Gal4, UAS-Dfz2-GFP* are responsive to further reductions in Wnt/Wg signaling. **(E)** *nub-Gal4, UAS-Dfz2-GPI UAS-wif1* showed similar effects as *nub-Gal4 UAS-Dfz2-GFP* alone, showing that expression of Dlp does not enhance of the effects of DFz2-GPI expression.
