# Supplementary material for: The Role of Glypicans in Wnt Inhibitory Factor-1 Activity and the Structural Basis of Wif1's Effects on Wnt and Hedgehog Signaling
Source: PLoS Genet. 2012 Feb 23;8(2):e1002503. doi: 10.1371/journal.pgen.1002503 (PMC3285576; doi:10.1371/journal.pgen.1002503)
Supplement: Figure S3 — Effects of Wif1 variants and Dlp on stout bristle differentiation. Expression of Dlp together with moderately driven pVal-wif1 or pVal-wif1ΔEGF genomic insertions produced additive reductions in bristle number. Due to slight variability in wing size, stout bristle number was normalized to the length of the sampling area defined in Arbitrary Units (A.U.) The sampling area began at the distal point where L2 intercepts the wing margin (see Figure 7A for L positions) and extended proximally along the anterior margin. The normalized stout bristle numbers were not different between the nub-gal4, pVal-UAS-wif1 and nub-gal4, pVal-UAS-wif1ΔEGF, UAS-dlp conditions. All other differences were statistically significant as determined by the two-tailed Mann-Whitney U Test (p<0.015). (PDF) [file pgen.1002503.s003.pdf]

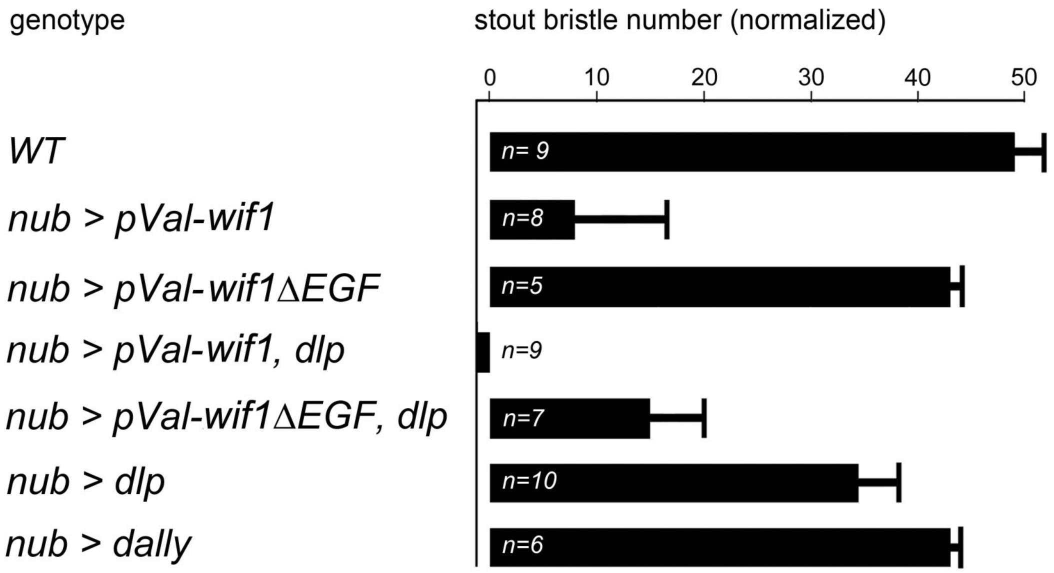

Figure S3. Effects of Wif1 variants and Dlp on stout bristle differentiation

Expression of Dlp together with moderately driven *pVal-wif1* or *pVal-wif1ΔEGF* genomic insertions produced additive reductions in bristle number. Due to slight variability in wing size, stout bristle number was normalized to the length of the sampling area defined in Arbitrary Units (A.U.) The sampling area began at the distal point where L2 intercepts the wing margin (see Figure 7A for L positions) and extended proximally along the anterior margin. The normalized stout bristle numbers were not different between the *nub-gal4, pVal-UAS-wif1* and *nub-gal4, pVal-UAS-wif1ΔEGF, UAS-dlp* conditions. All other differences were statistically significant as determined by the two-tailed Mann-Whitney U Test ($p<0.015$)
