# Supplementary material for: The Role of Glypicans in Wnt Inhibitory Factor-1 Activity and the Structural Basis of Wif1's Effects on Wnt and Hedgehog Signaling
Source: PLoS Genet. 2012 Feb 23;8(2):e1002503. doi: 10.1371/journal.pgen.1002503 (PMC3285576; doi:10.1371/journal.pgen.1002503)
Supplement: Figure S4 — Wif1 expression in zebrafish embryos leads to decreased expression of a β-catenin-regulated reporter. (A, A′) DIC (A) and fluorescence (A′) images of embryos carrying the Tg(TOP:dGFP) reporter, showing GFP expression in the dorsal midbrain (arrow), anterior to the midbrain-hindbrain boundary. (B) Injection of 40 pg/nL of wif1 mRNA reduces GFP expression (arrow). (C) Injection of 5 pg/nL of kny mRNA does not change GFP expression. (D) GFP intensities were measured in living embryos and their statistically significant differences were verified by two-tailed Mann-Whitney U test. Statistically significant differences in GFP intensity were measured. No differences of statistical significance from were observed between wild-type and kny-injected embryos. (PDF) [file pgen.1002503.s004.pdf]

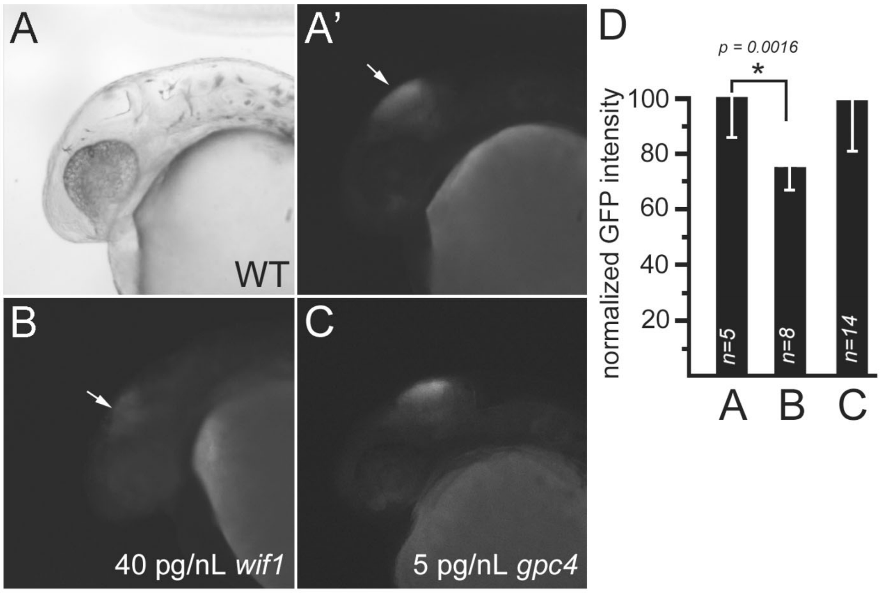

Figure S4. Wif1 expression in zebrafish embryos leads to decreased expression of a β-catenin-regulated reporter.

(A, A') DIC (A) and fluorescence (A') images of embryos carrying the *Tg*(*TOP:dGFP*) reporter, showing GFP expression in the dorsal midbrain (arrow), anterior to the midbrain-hindbrain boundary. **(B)** Injection of 40 pg/nL of *wif1* mRNA reduces GFP expression (arrow). **(C)** Injection of 5 pg/nL of *kny* mRNA does not change GFP expression. **(D)** GFP intensities were measured in living embryos and their statistically significant differences were verified by two-tailed Mann-Whitney U test. Statistically significant differences in GFP intensity were measured. No differences of statistical significance from were observed between wild-type and *kny*-injected embryos.
