# Supplementary material for: The Role of Glypicans in Wnt Inhibitory Factor-1 Activity and the Structural Basis of Wif1's Effects on Wnt and Hedgehog Signaling
Source: PLoS Genet. 2012 Feb 23;8(2):e1002503. doi: 10.1371/journal.pgen.1002503 (PMC3285576; doi:10.1371/journal.pgen.1002503)
Supplement: Figure S5 — Shf interactions with the D. melanogaster glypican Dally require normal ‘EGF-like’ domains. (A, B) Changes in anti-Shf staining (red) in wild type discs (WT) containing dally mutant clones (A) or overexpressing dally (B). Anti-Shf staining is reduced (asterisk) in a dally mutant clone (marked by the absence of GFP in green) in the anterior compartment (A). Dorsal, ap-Gal4-driven expression of UAS-dally (marked using UAS-GFP, green) stabilizes Shf (B, asterisk). (C, D) In shf2, discs, anti-Shf staining is not reduced in an anterior dally mutant clone (C, asterisk), or increased by dorsal, ap-gal4 driven UAS-dally expression (D, asterisk). Shf2 protein contains a missense mutation in Shf's third “EGF-like’ domain. (PDF) [file pgen.1002503.s005.pdf]

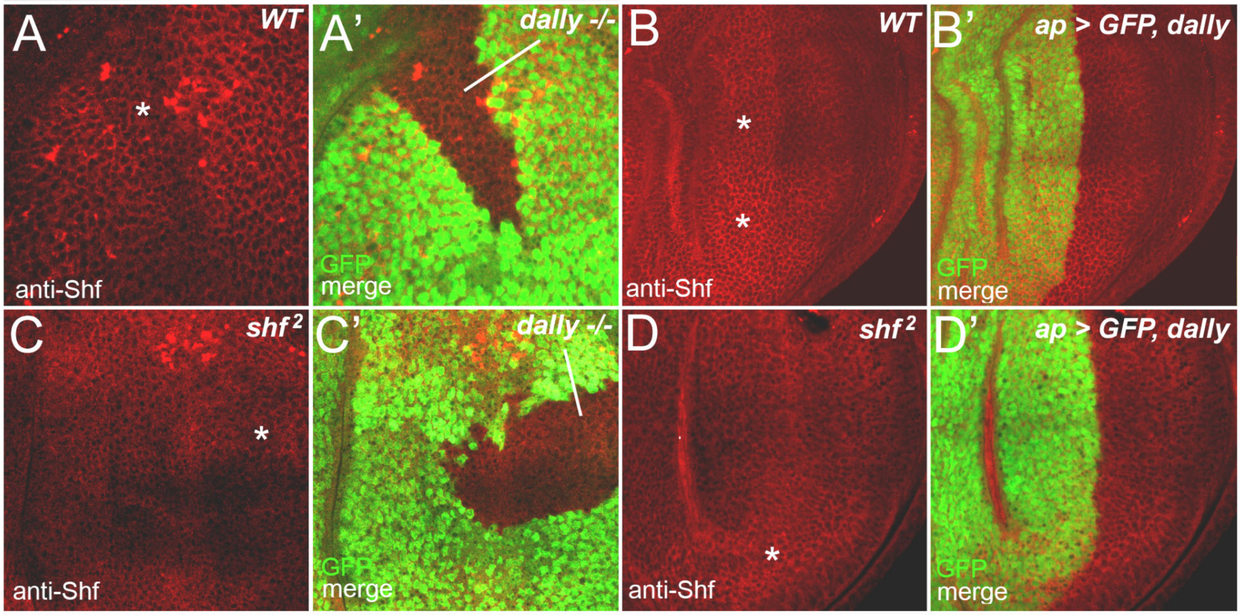

Figure S5. Shf interactions with the *D. melanogaster* glypican Dally require normal 'EGF-like' domains.

(A, B) Changes in anti-Shf staining (red) in wild type discs (WT) containing *dally* mutant clones (A) or overexpressing *dally* (B). Anti-Shf staining is reduced (asterisk) in a *dally* mutant clone (marked by the absence of GFP in green) in the anterior compartment (A). Dorsal, *ap-Gal4*-driven expression of *UAS-dally* (marked using *UAS-GFP*, green) stabilizes Shf (B, asterisk). **(C, D)** In *shf*^{2}, discs, anti-Shf staining is not reduced in an anterior *dally* mutant clone (C, asterisk), or increased by dorsal, *ap-gal4* driven *UAS-dally* expression (D, asterisk). Shf^2 protein contains a missense mutation in Shf's third "EGF-like' domain.
