# Supplementary material for: The Role of Glypicans in Wnt Inhibitory Factor-1 Activity and the Structural Basis of Wif1's Effects on Wnt and Hedgehog Signaling
Source: PLoS Genet. 2012 Feb 23;8(2):e1002503. doi: 10.1371/journal.pgen.1002503 (PMC3285576; doi:10.1371/journal.pgen.1002503)
Supplement: Figure S6 — Full length and EGF-depleted Wif1 are secreted by Drosophila S2 cells. Constructs were tagged with V5 epitope at their C-termini (Materials and Methods). Respective pVal-UAS-wif1-V5 and pVal-UAS-wif1ΔEGF-V5 were co-transfected with pAW-Gal4. Supernatants were harvested at day 5 post-transfection. The low molecular weight band represents Wif1ΔEGF (lane A), which is fully stable since it is secreted at levels virtually equal to the levels of the full length Wif1 (lane B). (PDF) [file pgen.1002503.s006.pdf]

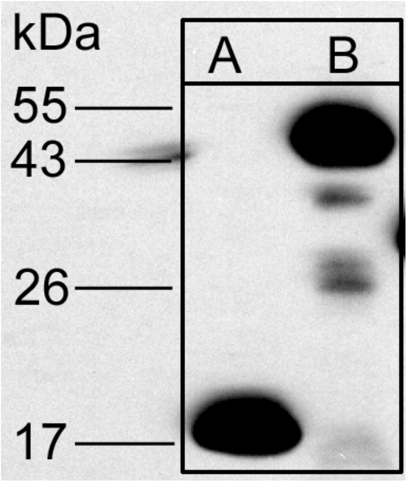

Figure S6. Full length and EGF-depleted Wif1 are secreted by *Drosophila* S2 cells

Constructs were tagged with V5 epitope at their C-termini (Materials and Methods). Respective *pVal-UAS-wif1-V5* and *pVal-UAS-wif1ΔEGF-V5* were co-transfected with *pAW-Gal4*. Supernatants were harvested at day 5 post-transfection. The low molecular weight band represents Wif1ΔEGF (lane A), which is fully stable since it is secreted at levels virtually equal to the levels of the full length Wif1 (lane B).
