# Supplementary material for: The Role of Glypicans in Wnt Inhibitory Factor-1 Activity and the Structural Basis of Wif1's Effects on Wnt and Hedgehog Signaling
Source: PLoS Genet. 2012 Feb 23;8(2):e1002503. doi: 10.1371/journal.pgen.1002503 (PMC3285576; doi:10.1371/journal.pgen.1002503)
Supplement: Figure S7 — Shf partially alleviates Wg signaling defects in UAS-wif1 or UAS-Dfz2-GPI expressing wings. (A, B) UAS-shf reduces notching defects in UAS-wif1 expressing wings, restoring L1 and some anterior bristles. (C, D) UAS-shf increases wing growth in UAS-Dfz2-GPI-expressing wings. Wings expressing UAS-shf alone are indistinguishable from wild-type [2], [3]. (PDF) [file pgen.1002503.s007.pdf]

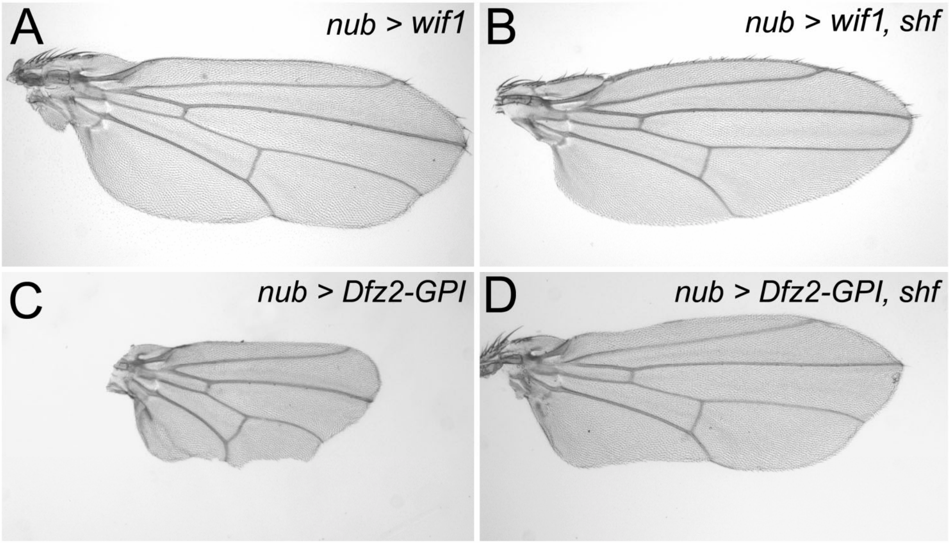

Figure S7. Shf partially alleviates Wg signaling defects in *UAS-wif1* or *UAS-Dfz2-GPI* expressing wings

(A, B) *UAS-shf* reduces notching defects in *UAS-wif1* expressing wings, restoring L1 and some anterior bristles. **(C, D)** *UAS-shf* increases wing growth in *UAS-Dfz2-GPI*-expressing wings. Wings expressing *UAS-shf* alone are indistinguishable from wild-type [2,3].
