# Supplementary material for: The Role of Glypicans in Wnt Inhibitory Factor-1 Activity and the Structural Basis of Wif1's Effects on Wnt and Hedgehog Signaling
Source: PLoS Genet. 2012 Feb 23;8(2):e1002503. doi: 10.1371/journal.pgen.1002503 (PMC3285576; doi:10.1371/journal.pgen.1002503)
Supplement: Figure S8 — The effects of Wg signaling on CiAct accumulation in shf mutants. (A) Strong inhibition of Wg signaling in a shfx33/Y wing disc, using nub-gal4- driven UAS-Dfz2-GPI, did not alter the width of the domain with strong accumulation of CiAct. Compare to anti-CiAct staining in shf mutants in Figure 6D. (B) nub-gal4-driven expression of UAS-wif1 in a shfx33/Y wing disc caused a broader, less intense domain of CiAct. (PDF) [file pgen.1002503.s008.pdf]

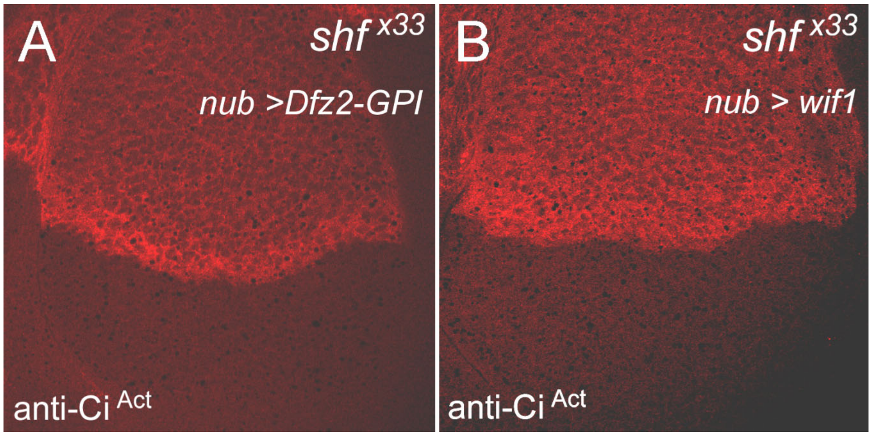

Figure S8. The effects of Wg signaling on Ci^{Act} accumulation in *shf* mutants

(A) Strong inhibition of Wg signaling in a *shf*^{x33}/*Y* wing disc, using *nub-gal4-* driven *UAS-Dfz2-GPI*, did not alter the width of the domain with strong accumulation of Ci^{Act}. Compare to anti-Ci^{Act} staining in *shf* mutants in Figure 6D. **(B)** *nub-gal4*-driven expression of *UAS-wif1* in a *shf*^{x33}/*Y* wing disc caused a broader, less intense domain of Ci^{Act}.
