# Supplementary material for: The Role of Glypicans in Wnt Inhibitory Factor-1 Activity and the Structural Basis of Wif1's Effects on Wnt and Hedgehog Signaling
Source: PLoS Genet. 2012 Feb 23;8(2):e1002503. doi: 10.1371/journal.pgen.1002503 (PMC3285576; doi:10.1371/journal.pgen.1002503)
Supplement: Figure S9 — Effects of Wif1 and chimeric Wif1:Shf proteins on Hh-GFP levels. Constructs were expressed in shf nulls (shfx33). The image in the upper panel shows areas sampled for the measurements, and the chart shows the GFP intensities in arbitrary units. Values obtained for the Posterior-Ventral quadrant (P–V) were normalized, and expressed as the percentage of signal intensity compared to the Anterior Ventral quadrant (A–V) where the Hh-GFP signal is at background levels. Graph shows averaged values and error bars show standard deviation. Differences of statistical significance were determined using the two-tailed Mann-Whitney U Test. In shf discs, Hh-GFP diffusion into the ventral compartment was significantly enhanced (asterisks) by Wif1 (p = 0.023) or WIFWif1-EGFShf (p<0.001), but not significantly enhanced by WIFShf-EGFWif1 (p = 0.153). (PDF) [file pgen.1002503.s009.pdf]

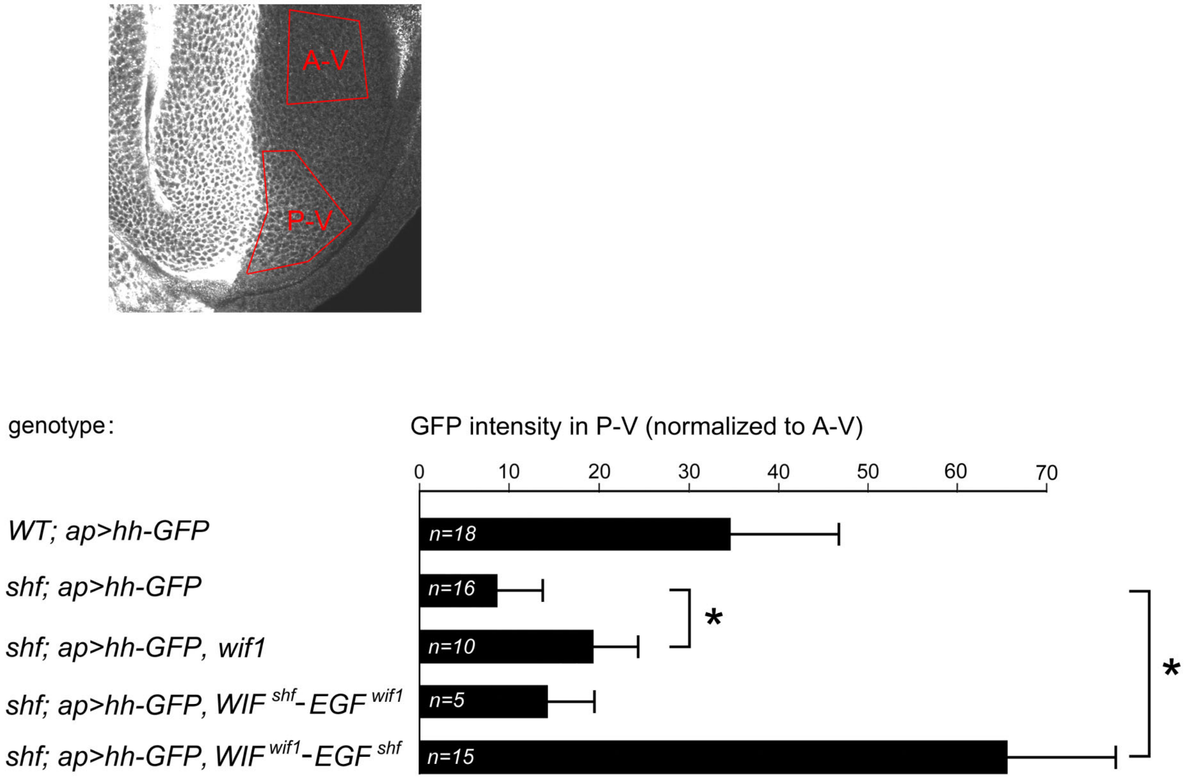

Figure S9. Effects of Wif1 and chimeric Wif1:Shf proteins on Hh-GFP levels

Constructs were expressed in *shf* nulls (*shf*^{x33}). The image in the upper panel shows areas sampled for the measurements, and the chart shows the GFP intensities in arbitrary units. Values obtained for the Posterior-Ventral quadrant (P-V) were normalized, and expressed as the percentage of signal intensity compared to the Anterior Ventral quadrant (A-V) where the Hh-GFP signal is at background levels. Graph shows averaged values and error bars show standard deviation. Differences of statistical significance were determined using the two-tailed Mann-Whitney U Test. In *shf* discs, Hh-GFP diffusion into the ventral compartment was significantly enhanced (asterisks) by Wif1 ($p=0.023$) or WIF^{Wif1}-EGF^{Shf} ($p<0.001$), but not significantly enhanced by WIF^{Shf}-EGF^{Wif1} ($p=0.153$).
